# Active BRAF-V600E is the key player in generation of a sessile serrated polyp-specific DNA methylation profile

**DOI:** 10.1371/journal.pone.0192499

**Published:** 2018-03-28

**Authors:** Somaye Dehghanizadeh, Vahid Khoddami, Timothy L. Mosbruger, Sue S. Hammoud, Kornelia Edes, Therese S. Berry, Michelle Done, Wade S. Samowitz, James A. DiSario, Daniel G. Luba, Randall W. Burt, David A. Jones

**Affiliations:** 1 Department of Oncological Sciences, Huntsman Cancer Institute, University of Utah School of Medicine, Salt Lake City, UT, United States of America; 2 Department of Cell Biology, Harvard Medical School, Boston, MA, United States of America; 3 Department of Stem Cells and Developmental Biology, Cell Science Research Center, Royan Institute for Stem Cell Biology and Technology, ACECR, Tehran, Iran; 4 Huntsman Cancer Institute, University of Utah School of Medicine, Salt Lake City, UT, United States of America; 5 Department of Human Genetics, University of Michigan Medical School, Ann Arbor, MI, United States of America; 6 Department of Pathology, University of Utah School of Medicine, Salt Lake City, UT, United States of America; 7 The Monterey Bay Gastroenterology Research Institute, Monterey, CA, United States of America; 8 Department of Internal Medicine, University of Utah School of Medicine, Salt Lake City, UT, United States of America; 9 Functional and Chemical Genomics, Oklahoma Medical Research Foundation, Oklahoma City, OK, United States of America; Howard University, UNITED STATES

## Abstract

**Background:**

Sessile serrated polyps (SSPs) have emerged as important precursors for a large number of sporadic colorectal cancers. They are difficult to detect during colonoscopy due to their flat shape and the excessive amounts of secreted mucin that cover the polyps. The underlying genetic and epigenetic basis for the emergence of SSPs is largely unknown with existing genetic studies confined to a limited number of oncogenes and tumor suppressors. A full characterization of the genetic and epigenetic landscape of SSPs would provide insight into their origin and potentially offer new biomarkers useful for detection of SSPs in stool samples.

**Methods:**

We used a combination of genome-wide mutation detection, exome sequencing and DNA methylation profiling (via methyl-array and whole-genome bisulfite sequencing) to analyze multiple samples of sessile serrated polyps and compared these to familial adenomatous polyps.

**Results:**

Our analysis revealed BRAF-V600E as the sole recurring somatic mutation in SSPs with no additional major genetic mutations detected. The occurrence of BRAF-V600E was coincident with a unique DNA methylation pattern revealing a set of DNA methylation markers showing significant (~3 to 30 fold) increase in their methylation levels, exclusively in SSP samples. These methylation patterns effectively distinguished sessile serrated polys from adenomatous polyps and did so more effectively than parallel gene expression profiles.

**Conclusions:**

This study provides an important example of a single oncogenic mutation leading to reproducible global DNA methylation changes. These methylated markers are specific to SSPs and could be of important clinical relevance for the early diagnosis of SSPs using non-invasive approaches such as fecal DNA testing.

## Introduction

Colorectal cancer (CRC) is the third most common diagnosed and the fourth death causing cancer worldwide and causes about 600,000 deaths every year (WHO 2012). Mutations in APC, KRAS, BRAF, TP53, and TGF signaling genes are detected frequently in CRCs [1, 2]. While about 5% of CRCs show a family history, the majority of CRCs happen sporadically. Recent evidence suggests that one third of sporadic colorectal cancers are thought to form from the progression of premalignant serrated lesions [3, 4]. Serrated lesions are classified into three categories; hyperplastic polyps (HP), sessile serrated adenomas/polyps (SSA/P), and traditional serrated adenomas (TSA). The basis for this classification is the distinct cyto-histological features such as morphology and architecture of colon crypts, as well as position and extent of the proliferative zone in the crypts [3–10]. However, the underlying molecular pathways and possible genetic and epigenetic differences generating such distinctions are largely unknown. Of these three categories HP shows the highest frequency in population but not appears to advance to carcinoma. In contrast both SSP and TSA show significant malignant potential to develop into cancer. However, the much higher incidences of SSP in population, in comparison to TSA, makes SSP the principal precursor of colorectal cancers derived from serrated lesions [3].

BRAF (V-Raf Murine Sarcoma Viral Oncogene Homolog B) is a serine/threonine protein kinase functioning downstream of the KRAS in the MAPK signaling pathway, with roles in cell proliferation and differentiation. BRAF is mutated in 5–10% of all malignancies (e.g. melanoma 60–80%, colorectal cancer 5–20%, and papillary thyroid cancer 30–50%) and is associated with changes in DNA methylation[11–14]. Mutation of valine at residue number 600 to glutamic acid (BRAF-V600E), an activation mutation, constitutes more than 90% of all BRAF mutations[15]. Previous surveys of cancer associated mutations in SSP samples, through targeted analysis of limited known mutations identified the BRAF-V600E as the key mutation in this disease[3, 16, 17]. However, it is not clear whether other mutations in the same samples contribute to the etiology of this disease.

The BRAF-V600E and mutation in codon 12 and 13 of KRAS in mouse intestine are shown to develop serrated lesions, that after deletion of P16 lead to tumorigenesis[18–20]. In human, both BRAF and KRAS mutations are reported to be associated with serrated polyps. According to a recent expert panel, however, SSP is mostly associated with BRAF mutation and the KRAS mutation is linked to TSA [3].

There are subtypes in almost all cancer types that show, in selected CpG islands, a gain of methylation phenotype known as CpG island methylator phenotype (CIMP). CIMP was initially described in CRCs and colon adenomas[21] and has been widely used for characterization of DNA methylation status of other cancer types. About ten specific regions of DNA are currently tested for gain of methylation in defining CIMP-positive CRCs[22–25]. Previous studies on SSP showed that SSP harbor DNA methylation at these regions and is, therefore, classified as a CIMP-positive [3, 17, 22–25].

Given the importance of SSP in colon cancer development, there is an urgent need to understand its detailed molecular features at the genetic and epigenetic levels. Here we apply state-of-the-art technologies to further characterize SSPs as the most predominant premalignant serrated lesions. We show that BRAF-V600E is the sole recurrent somatic mutation associated with SSP. And that SSPs harbor a unique DNA methylation profile associated with BRAF-V600E that may be applicable for molecular characterization of SSP in diagnostic procedures.

## Materials and methods

These studies were approved by the University of Utah Institutional Review Board. Written consent was obtained from all participants. No minors were involved.

### Sample collection and preparation

All patient samples except P7-SSP-9 and P7-TSA-1 (TSA; traditional serrated adenoma) were collected as biopsies in Huntsman Cancer Hospital. SSP-9 and TSA-1 are provided by the Monterey Bay Gastroenterology Research Institute in FFPE blocks. P1-SSP-1, P1-SSP-2 and P1-SSP-3 were fixed in 10% formaldehyde and embedded in paraffin. Other biopsy samples were flash frozen in liquid nitrogen, and later embedded in O.C.T. (optimal cutting temperature compound). Five micron sections were cut from both FFPE and O.C.T. blocks using Leica microtome or Leica cryostat respectively. The hematoxylin and eosin (H&E) stained sections were used for characterization based on histopathological features. All patients provided written consent for the proposed studies. No minors were included in the patient group.

### Whole-exome sequencing

The characterized biopsies were subjected to sample isolation from the affected areas of the blocks using 1mm diameter disposable biopsy punches (Miltex). The QIAamp DNA FFPE Tissue kit (Qiagen) was used to extract DNA from FFPE tissues. The Qiagen AllPrep kit was used to extract both DNA and RNA from frozen O.C.T. embedded tissues. DNeasy Blood & Tissue kit (Qiagen) was used to extract DNA from blood samples. Library construction for exome sequencing was performed using Agilent Technologies SureSelect XT Reagent Kit, HSQ as described below. Briefly, 1–3 μg genomic DNA was sheared using Covaris S2 Focused-ultrasonicator, in a 130 μl volume, with 5.0 intensity, 10% duty cycle, 200 cycles per burst, and 6:00 minutes treatment time. Sheared DNA was purified using QIAquick PCR Purification kit (Qiagen). Using a combination of enzymes with filling and exonuclease activity, the DNA fragments became blunt ended. To prepare the fragments for accepting adaptors and to block concatamerization, an A overhang was added to the 3’ end of the fragments, followed by ligation to adaptors with T overhangs. Adaptor-ligated fragments were amplified using 6 cycle PCR and followed by PCR product purification using Agencourt AMPure XP beads. Concentrations and fragment size ranges of the libraries were measured using NanoDrop spectrophotometer and D1K ScreenTape assay in the Agilent 2200 TapeStation system. Exome capture was performed on about 750 ng of the amplified libraries via biotinylated RNA bait molecules of SureSelect Human All Exon 50Mb (Agilent). The captured molecules were purified using Dynabeads MyOne Streptavidin T1, and index tagged in 10 PCR cycles. The amplified exome-enriched fragments were purified on Agencourt AMPure XP beads. Concentration and size measurements were done using Invitrogen Qubit dsDNA HS Assay and High Sensitivity D1K assay. The KapaBiosystems Kapa Library Quant kit was used to quantitate the concentration of adaptor ligated libraries. The captured libraries were sequenced using HiSeq 2000 101 cycle paired-end sequencing. The exome sequencing datasets are publicly available through GEO with the following accession number: GSE110535.

Sequenced reads were aligned to the human genome (hg19) using novoalign (http://www.novocraft.com) (2.08.1). SAMtools[26] (0.1.18) was used to create mpileup files from the alignments. VarScan (2.3.5) was used for finding the germline and somatic mutations in polyps through comparison with the data from paired blood samples, and also the hg19 reference genome. Variations with frequency of >5% in polyps were considered in the analysis. The variations found using VarScan were annotated using Annovar[27] with addition of the data from 1000 Genomes project, dbSNP 121, TFBS, DGV (Database of Genomic Variants), SIFT, and PolyPhen-2.

### Mutation verification

The BRAF-V600E mutation and mutations at KRAS codon 12 and 13, were specifically tested by PCR amplification and sequencing, using the primer sets listed in **S1 Table**.

### DNA methylation profiling by methyl-array

Bisulfite conversion of 500 ng genomic DNA (extracted from patient samples (P1-SSP-3, P2-SSP-4, P5-SSP-7, P6-SSP-8, P7-SSP-9, P7-TSA-1, P8-TSA-2, P8-FAP-1, P9-FAP-2, P10-FAP-3, P11-Ca-1, P12-Ca-2, P1-GU-1, P13-NA-1, and P13-NS-1) was performed using EZ DNA methylation kit (Zymo Research) according to the manufacture protocol with few modifications, recommended by Illumina specifically for 450K methyl-array, including the incubation of the mixture of DNA and M-dilution buffer at 42°C for 30 minutes and also after addition of CT conversion reagent they went through 16 cycles of 30 seconds denaturation at 95°C followed by an hour incubation at 50°C. Converted DNA was hybridized to the HumanMethylation450 Analysis BeadChip using the Illumina Infinium 450K Methylation assay protocol. The arrays were imaged on the Illumina iScan instrument. GenomeStudio software v2011.1 (Illumina) was used to view the controls, calculate the Beta values for each CpG sites and make the hierarchical clustering by correlation metrics. The Beta value report from GenomeStudio was analyzed for differential methylation using Partek Genomic Suite (version 6.12.1227). The methyl-array outputs are publicly available through GEO with following accession.

### Whole-genome bisulfite sequencing

Three micrograms of genomic DNA (extracted from P1-SSP-3, P1-GU-1 and P1-blood) were sheared using Covaris S2 Focused-ultrasonicator, in a 130 μl volume, with 5.0 intensity, 10% duty cycle, 200 cycles per burst, and 6:00 minutes treatment time. Sheared DNA was purified using QIAquick PCR purification kit. Unmethylated DNA of lambda phage was added at 1% concentration to each sample as an internal control of monitoring the conversion efficiency. Libraries were made according to the Illumina library preparation protocol using the methylated adaptors. The libraries were purified using QIAquick PCR purification kit and bisulfite converted using EpiTect bisulfite kit (Qiagen). To increase the conversion efficiency we performed the denaturation steps at 98°C for 10 minutes, and the samples were incubated for another 120 minutes at 60°C at the end of thermocycling reaction. The bisulfite converted libraries were amplified using PfuTurbo HotStart DNA Polymerase (Agilent) for 16 cycles. The libraries were then size selected (200-300bp) and gel purified. The quality of libraries were checked using DNA 1000 Bioanalyzer chip. Libraries were then quantitated by qPCR using KapaBiosystems Kapa Library Quant kit and were run through 101 paired-end Illumina HiSeq 2000. The whole genome bisulfite sequencing datasets are publicly available through GEO with the following accession number: GSE110537.

Fastq Illumina sequencing reads were aligned to the human genome (hg19) using Novoalign (Novocraft). All downstream analysis were processed by programs in USeq package (http://useq.sourceforge.net). NovoalignBisulfiteParser was used to pars the text file from Novoalign into point data for each cytosine in the genome. The point data was parsed into CpG context using ParsePointDataContexts. The fraction methylation of each cytosine, or each CpG, with minimum 8 reads was calculated by BisStat. To calculate the FDR, BisStat used the fraction of non-converted cytosines from control lambda genome. BisStat calculated fraction methylation for windows of 1000 bp that contain minimum of 5 CpGs to find all methylated regions over the genome, with maximum FDR of 0.01, and made outputs to visualize on IGB. To focus on a particular region FilterPointData was used to filter the point data for the regions of interest. The output was then used by BisStat to find fraction methylation on that particular region. BisSeq was used to find the DMRs of SSP compared to GU. It scanned the parsed CpG point data with window of 200 bp that has minimum 5 CpGs differentially methylated with 3 fold changes and maximum FDR of 0.001. BisSeqAggregatePlotter was used to plot class average aggregate of fraction methylation from CpG point data over the regions of interest. BisStatRegionMaker was used to extract all the regions that are methylated from the BisStat’s serialized window objects. IntersectRegions was used to intersect two region files and calculate the correlation p-value.

The mean base fraction methylation over 10 Mb intervals of hg19 were plotted using Circos (v0.64) (http://circos.ca/), and the mean base fraction methylation of specific regions were plotted in R (v3.0.1) using ggplot2 (v0.9.3.1).

### DNA methylation verification

A selection of methylation data from WGBS and methyl-array was validated by bisulfite PCR sequencing. The primers are listed in **S2 Table**.

### Gene expression profiling by RNAseq and analysis

RNA was extracted from O.C.T embeded frozen colon biopsies (P1-SSP-3, P2-SSP-4, P5-SSP-7, P6-SSP-8, P8-TSA-2, P9-FAP-2, P10-FAP-3, P11-Ca-1, P12-Ca-2, P1-GU-1, P13-NA-1, P13-NS-1, P14-NS-2, P15-NA-3) as explained above. Total RNA was DNase treated using TURBO DNA-free Kit (Ambion), and its quality was assessed on Bioanalyzer RNA 6000 Nano Chip. For ribosomal RNA depletion, the total RNA was treated with RiboMinus kit (Life Technologies), then directional RNAseq library was performed according to Illumina’s protocol. Libraries were sequenced using 50bp single-end format on Illumina HiSeq 2000. Sequenced reads were aligned to the human genome (hg19) plus extended splice junctions. The USeq (8.4.6) package [28] was used to convert spliced alignments back to genomic space and generate per gene read counts. The read counts were used in the DESeq (1.12.0) and EdgeR (3.2.4) bioconductor packages to determine differential gene expression. DESeq first normalizes the data by dividing raw counts for each sample by the median of the per-gene geometric means of the raw counts across all samples. DESeq then tests for differential expression using the negative binomial distribution and a shrinkage estimator for the distribution’s variance [29]. Genes with absolute log2 ratios >1 and FDR <0.01 were considered significant. EdgeR first calculates a normalization factor using the trimmed mean of log2 fold change (TMM) method. Dispersions were then estimated using quantile-adjusted conditional maximum likelihood (qCML). Finally, differential expression was determined using an exact test for the negative binomial distribution [30]. Genes with an absolute log2 ratio >1 and FDR <0.01 were considered significant. The Gene expression profiling datasets are publicly available through GEO with the following accession number: GSE110536.

## Results

### BRAF-V600E is the sole recurring somatic mutation in SSPs

Previous studies have shown, via targeted mutation analysis of candidate markers, that BRAF-V600E is the major mutation underlying SSP. In addition, KRAS mutations predominantly at codons 12 and 13 have also been assigned to SSP, although later studies have connected the KRAS mutations to the TSA type polyps instead [3]. Overall, due to lack of comprehensive genome-wide mutation analysis surveys, involvement of additional mutation(s) underlying SSP is still largely unclear. To address this we performed exome sequencing on DNA extracted from total of eight SSP samples from six patients diagnosed, via colonoscopy and pathological inspection of polyp sections, to have typical SSP type polyps in their colons. From five of these patients a single polyp was analyzed to study the common mutations between different affected individuals. From the sixth patient three different polyps were isolated from different locations of the colon, providing the opportunity to separately sequence the DNA extracted from all three samples to study the possible differences between different polyps of the same individual. In parallel to these eight SSP samples, exome sequencing was performed on corresponding available paired blood samples (from four patients; including the one with three different polyps) to filter the germline mutations and single nucleotide polymorphisms (SNPs).

For the exome sequencing we used the SureSelect Human All Exon system (Agilent), covering about 51 million bases of the whole genome. This system is designed to capture all known and predicted exonic sequences of about 21,000 genes from both of protein coding and non-coding types. The released DNA fragments were then subjected to library preparation followed by sequencing by HiSeq-2000 in a 101bp paired-end sequencing format. Sequencing reads were aligned to H. sapiens Feb 2009 genome build (hg19) using the commercial Novoalign package revealing about 6.87 Gb mapped deduplicated reads, on average, for each sequenced sample, from that 3.59 Gb are on target. On average 92%, 80%, and 68% of the exome are covered with more than 10, 20, or 30 sequenced reads, respectively (**S3 Table**). To assess different variant types (e.g. SNP, insertion and deletion), the sequenced exomes (including the SSP-Blood paired data series) were compared to the reference genome using VarScan package [31]. Between 17 to 30 thousand variations were detected in exonic regions and splicing junctions of all sequenced samples (**S4 Table**). To narrow down the list of variations into disease-associated mutations and isolate the relevant mutations, we used four criteria to filter the list of variations. Specifically, we excluded: 1) all synonymous mutations (mutations in coding sequence that do not alter the amino acid sequence), 2) variations reported by the 1000 genome project with the frequency of > 0.05 in the database—with the assumption that this frequency is much higher than the estimated SSP frequency in the human population, 3) variations that are in duplicated regions of the genome because of the redundancy, and 4) mutations with PolyPhen-2 arbitrary cutoff of < 0.1 (PolyPhen-2 (http://genetics.bwh.harvard.edu/pph2/) predicts the functional effect of the mutation based on multiple alignment and the conserved regions of protein and also the structural data). Finally, to identify the SSP-associated mutation(s), we looked for common non-synonymous somatic mutations that are present in all eight SSP samples. Notably, BRAF-V600E was the only common somatic mutation present in all eight SSP samples, including the three samples analyzed from the single individual, although this mutation was not detected in the grossly uninvolved (GU) biopsy of this patient. Interestingly, similar analysis of different combinations of smaller sample numbers (e.g. combinations of 7, 6, 5, or 4 samples) complemented by PCR verification resulted in the same finding; isolation of BRAF-V600E as the sole common mutation in all different combinations tested (**S5 Table**).

### The BRAF-V600E mutation correlates with a reproducible unique DNA methylation signature

Previous studies classify SSP as a CIMP-positive polyp showing gain of methylation in a limited (<10) number of tested CpG islands (CIMP markers). However, a high-resolution full picture of the SSP-specific DNA methylation profile considering the complete list of differentially methylated regions, in comparison to normal or other colon precancerous states, is lacking due to lack of high-throughput DNA methylome studies. Importantly the possible connection between SSP-specific mutation (BRAF-V600E) and such SSP-specific DNA methylation profile is currently unknown. To this end, we performed genome-wide DNA methylation profiling on fifteen available colon biopsies from eleven individuals including five SSP, two TSA, three FAP, two carcinoma, one grossly uninvolved (GU) and two normal tissue samples. From these a single tissue sample was assessed per individual for seven patients, and from the other four individuals a pair of two samples per person was used including; 1) a SSP-GU pair, 2) a TSA-FAP pair, 3) a SSP-TSA pair, and 4) an NA-NS pair (two normal samples from the ascending (NA) and the sigmoid (NS) section of the colon) (Table 1 and S1 Fig).

**Table 1:** The biopsy samples. Mutations known for each sample are listed. SSP-1 to SSP-8 are exome sequenced. Mutations in other samples are tested by OncoCarta3. BRAF-V600E mutation and KRAS codon 12 and 13 mutations are tested by PCR-sequencing in all samples.

	SSP	TSA	FAP	Carcinoma	Normal
Patient #1	**SSP-1** (BRAF-V600E) **SSP-2** (BRAF-V600E) **SSP-3** (BRAF-V600E)				**GU-1**
Patient #2	**SSP-4** (BRAF-V600E)				
Patient #3	**SSP-5** (BRAF-V600E)				
Patient #4	**SSP-6** (BRAF-V600E)				
Patient #5	**SSP-7** (BRAF-V600E)				
Patient #6	**SSP-8** (BRAF-V600E)				
Patient #7	**SSP-9** (BRAF-V600E)	**TSA-1**			
Patient #8		**TSA-2** (BRAF-V600E; APC-Q1062fs*1)	**FAP-1** (APC-Q1062fs*1)		
Patient #9			**FAP-2** (APC-R1450*)		
Patient #10			**FAP-3** (APC-E1309fs*4)		
Patient #11				**Ca-1** (BRAF-V600E; APC-S1465fs*3; JAK3- V722I; PTEN- K267fs*9)	
Patient #12				**Ca-2**	
Patient #13					**NA-1, NS-1**
Patient #14					**NS-2**
Patient #15					**NA-3**

Characterization of the sample types was performed entirely based on the histopathological features of the hematoxylin and eosin (H&E) stained sample sections. To evaluate the genetic background of the samples regarding SSP-related mutations, all samples were separately subjected to mutation-detection analysis, via PCR amplification coupled with Sanger sequencing, specifically for the BRAF-V600E mutation and also the KRAS codon 12 and 13 mutations, the two mutations previously reported to have connection with SSP. This survey confirmed BRAF-V600E mutation in all SSP samples and also in one of the carcinoma samples and one of the TSA samples. Interestingly, the BRAF-V600E positive TSA sample was from the TSA-FAP pair, in which the FAP sample (isolated from a different section of the same polyp as the TSA sample) showed APC but not the BRAF-V600E mutation. This TSA-FAP sample pair provided us with a unique opportunity to study the effect of two distinct colon cancer-related mutations on the DNA methylation pattern, in a single polyp that provides the same genetic background and substantially identical tumor microenvironments. As a consequence, we also include FAP adenomas for comparison sake. Beside SSP-related mutations, to search for other possible cancer relevant mutations in the samples OncoCarta, a mutation detection panel based on mass-spectrometry (the Sequenom technology) was applied. Additionally, the FAP samples were specifically tested by sequencing of APC gene. The results of mutation-detection analysis for all tested samples are summarized in Table 1.

After defining the background mutation(s) in all samples, they were subjected to bisulfite treatment (using Zymo EZ DNA methylation kit) followed by DNA methylation analysis by Infinium 450K methyl-array (Illumina). This methyl-array works based on hybridization and single nucleotide extension, and is designed to survey total of 485,577 CpGs from the human genome. These CpGs are distributed over about 99% of the RefSeq genes (including the gene bodies, 5’ and 3’ UTRs and up to 1.5Kb upstream of the TSSs), and about 94% of the characterized CpG islands (CGIs) and their flanking first 2Kb (CGI shores) and second 2Kb (CGI shelves). We note here that for more accurate results we limited our analysis to only ~82% (395,899) of the CpGs on the methyl-array as the rest show overlap with known SNPs in the human genome causing errors in interpretation. However, as the excluded CpGs are almost evenly distributed over the entire genome their removal from the list will likely have minimal impact on the analysis.

**Fig 1:**
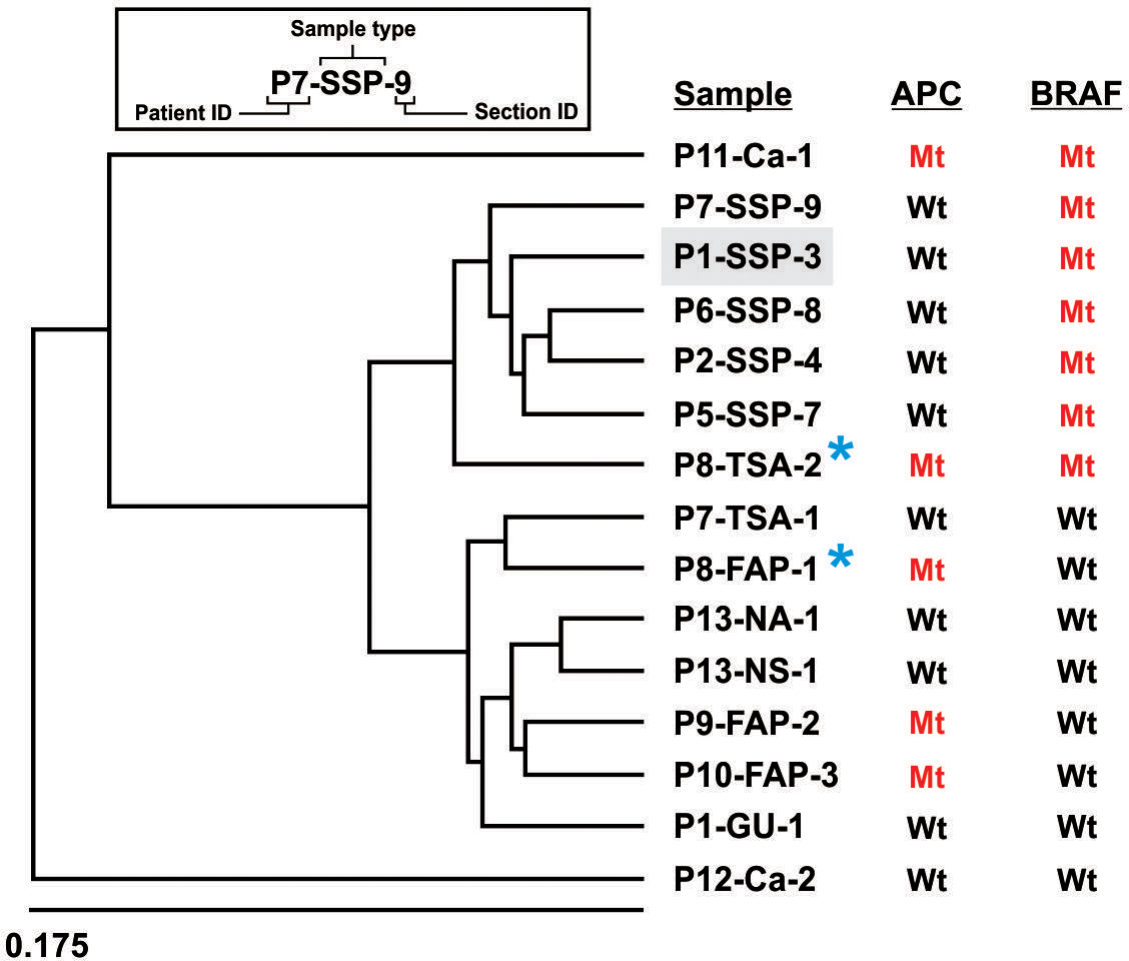
Hierarchical clustering of all CpGs in 450K methyl-array on colon samples. The SSP samples are clustered together based on DNA methylation. The TSA-2 sample that is mutant for BRAF gene also clustered with SSPs.

The data generated by methyl-array, for all samples, were preliminarily processed by GenomeStudio software (Illumina). This package was used for calculating the methylation indices for each CpG site, and for creating the hierarchical clustering of all samples collectively, to arrange them into distinct groups entirely based on their global methylation patterns. The main purpose of this analysis was to see whether different SSP samples show similar methylation patterns when analyzed in a pool with other malignant, premalignant and normal colon samples. As illustrated in Fig 1 the unbiased hierarchical clustering resulted in clustering of all SSP samples together, indicating the existence of a reproducible SSP-specific methylation pattern in different individuals.

Interestingly the BRAF-V600E positive TSA and carcinoma samples (both also containing mutation in the APC gene) clustered closely with SSP, but not FAP or other sample types. This strongly connects the BRAF-V600E activation mutation to a reproducible unique DNA methylation signature.

**Fig 2:**
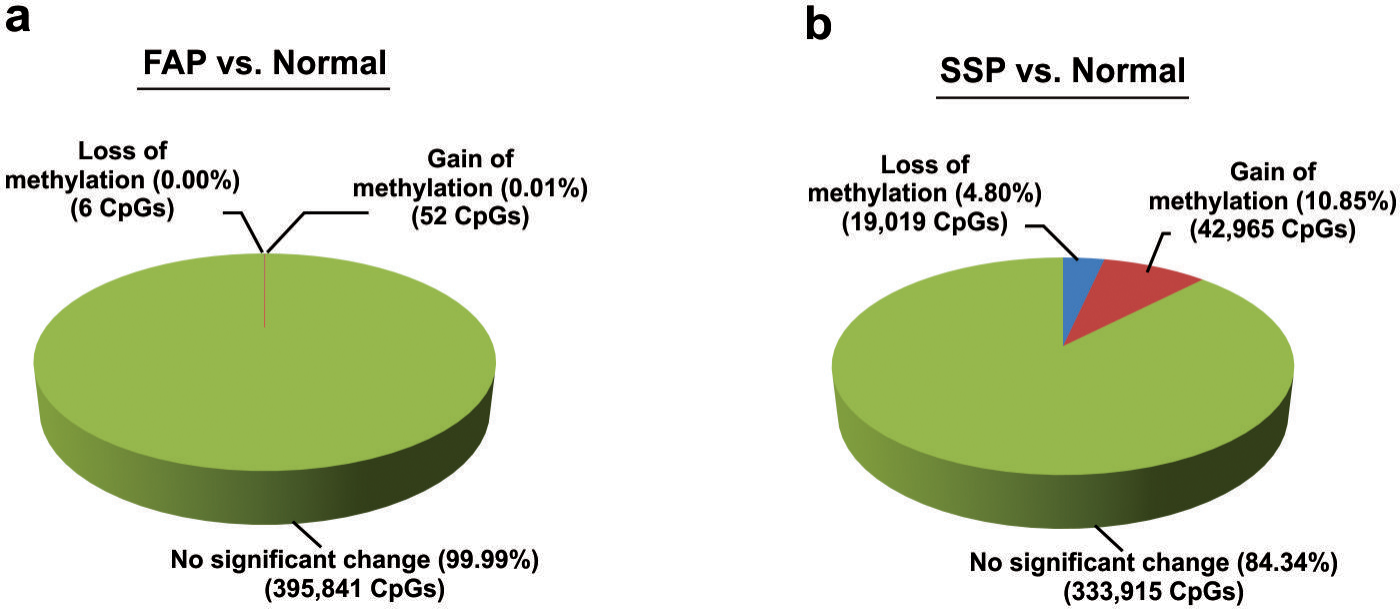
Global methylation changes in SSP and FAP. **(a)** Comparison of FAP with normal samples shows only few DNA methylation changes. From 395,899 CpGs tested on the Infinium array, only 52 CpGs show more than 2 fold increase in methylation in FAP compared to normal colon tissue and 6 CpGs show more than 2 fold reduction in methylation (p-value <0.05). **(b)** In contrast comparison of SSP with normal samples shows significant DNA methylation changes. From 395,899 CpGs tested on the Infinium array, 42,965 (10.85%) show more than 2 fold increase in methylation in SSP compared to normal colon tissue and 19,019 (4.80%) show more than 2 fold reduction in methylation (p-value <0.05).

To understand the SSP-specific DNA methylation signature, we performed differential methylation analysis, to define the gain and loss of methylation at each particular CpG site. For this purpose, we applied Genomic Suite package (Partek), to compare the methylation indices from each group of samples to the two normal samples. Comparison of SSP to normal datasets revealed 42,965 CpGs with gain and 19,019 CpGs with loss of methylation, equal to 10.85% and 4.80%, respectively (Fig 2A). In contrast the total number of CpGs showing methylation changes in FAP samples was only 58 (52 CpGs with gain and 6 with loss of methylation) from a total number of 395,899 CpGs tested (Fig 2B).

### The DNA methylation signature represents specific markers for molecular characterization of SSP

**Fig 3:**
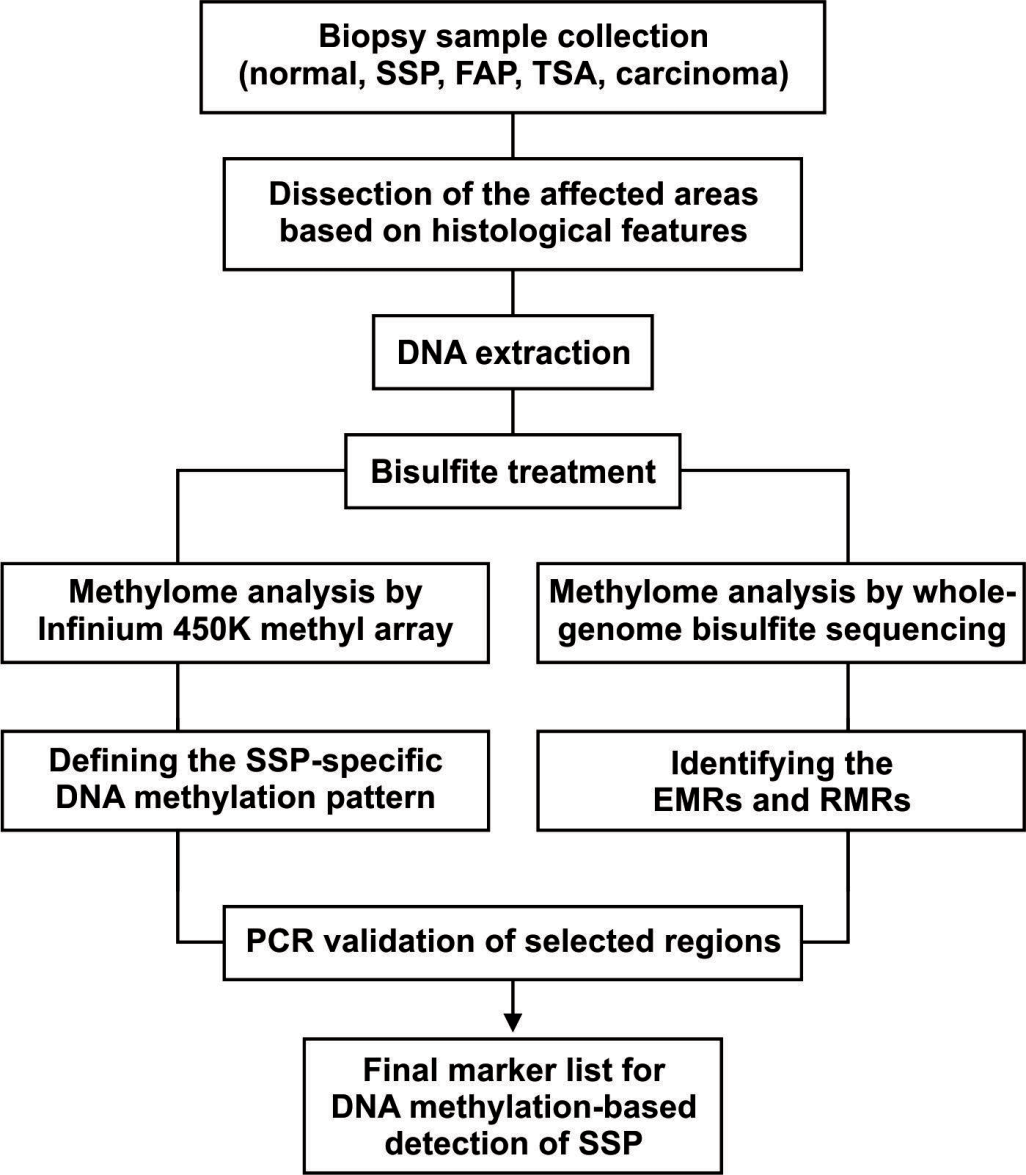
The workflow to determine the SSP-specific DNA methylation markers. The workflow shows the steps from sample collection to determination of the SSP-specific DNA methylation markers.

For further validation of the methyl-array results with another technology, and to provide a comprehensive high-resolution SSP-specific DNA methylome from the entire genome, we analyzed one of the SSP samples (P1-SSP3), paired with the grossly uninvolved (GU) colon sample and the blood sample from the same patient, with the whole-genome bisulfite sequencing (WGBS) approach. The DNA from these samples were subjected to bisulfite treatment followed by 101bp paired-end high-throughput sequencing. The sequencing datasets were then analyzed with USeq package. The detailed genome-wide analysis of the WGBS results for SSP, GU and blood samples are presented the section below. Here, in this section, to find the SSP-specific DNA methylation markers, we used the WGBS results to isolate the regions of the genome with significant gain of methylation, the enriched methylated regions (EMRs), exclusively in the SSP sample by comparing the methylation status of SSP to the paired GU. To list the SSP-specific EMRs we considered only EMRs with >10 folds gain of methylation with FDR < 0.001 in the SSP sample. To ensure the reproducibility, all SSP-specific EMRs obtained from this single SSP sample were then manually compared with the methyl-array results of other SSP samples. Those EMRs, in which their CpGs show significant gain of methylation (have > = 5 CpG on the array, average gain of methylation >10 folds, and FDR < 0.05) in all tested SSP samples with methyl-array were reported as SSP-specific DNA methylation markers for molecular characterization of SSPs. We note here that selection of the WGBS output for generation of the initial marker-list was because of the higher resolution of this approach in isolation of the regions harboring several CpGs with gain of methylation. Especially, with WGBS the CpGs in a single DNA molecule (a single sequencing read) are analyzed together while the methyl-array reports values for isolated individual CpGs. Regardless, for further validation of the final marker list, selected EMRs (markers) were evaluated by bisulfite treatment followed by PCR amplification and Sanger sequencing. The workflow is presented in Fig 3. The marker list is presented in Table 2.

**Table 2:** DNA methylation markers for SSP characterization and early diagnosis.

Marker ID (a)	Coordinate (b)	Distance from TSS (c)	Fold change (d)	FDR
**MAP6**	chr11:75378716–75380145	20	30	1.9 x 10–127
**EOMES**	chr3:27763797–27764601	2	18.3	7.9 x 10–20
**INSM2**	chr14:36003398–36004093	498	18.3	9.9 x 10–58
**PDGFD**	chr11:104034199–104034358	556	17.9	2.8 x 10–43
**PREX1**	chr20:47443621–47444377	420	17.1	4.2 x 10–77
**ARHGAP20**	chr11:110581500–110582693	676	15.7	5.3 x 10–55
**BMP3**	chr4:81951793–81953122	339	15.4	3.8 x 10–55
**SYNM**	chr15:99645330–99646614	687	14.6	3.2 x 10–100
**TRANK1**	chr3:36986263–36986757	37	14.5	1.4 x 10–63
**GUCY1A2**	chr11:106889458–106890171	565	14.5	1.2 x 10–135
**FBXO27**	chr19:39522128–39523340	430	14.3	2.4 x 10–49
**EPB41L3**	chr18:5628896–5630029	473	14.3	1.1 x 10–89
**CBS**	chr21:44494676–44496031	587	13.7	2.1 x 10–68
**FLT3**	chr13:28674052–28674910	225	13.7	4.5 x 10–65
**ELMO1**	chr7:37487274–37487986	828	13.4	9.2 x 10–39
**NPR1**	chr1:153651493–153652565	917	12.8	7.0 x 10–47
**NTNG2**	chr9:135036756–135037284	313	12.7	1.1 x 10–57
**GBX1**	chr7:150864018–150864478	386	12.7	5.4 x 10–21
**NKAIN2**	chr6:124123959–124124509	1051	12.5	7.6 x 10–62
**WNT5A**	chr3:55521138–55521632	55	12.2	2.8 x 10–38
**VWC2**	chr7:49812570–49813401	271	12.1	2.4 x 10–33
**NELL2**	chr12:45270250–45270997	9	12	7.2 x 10–54
**KCNK13**	chr14:90526615–90528500	550	11.8	5.8 x 10–55
**ODZ4**	chr11:79149255–79150246	493	11.7	7.5 x 10–98
**SCARF2**	chr22:20791428–20792147	324	11.5	3.1 x 10–21
**TBX2**	chr17:59477781–59478534	432	11.2	2.5 x 10–67

(a) The marker ID is designated based on the name of the gene in the proximity of the methylated region. For the complete list of markers see the **S1 File**.

(b) The precise coordinate of the differentially methylated region. The specified regions show >10 fold gain of methylation (p-value <0.001) in P1-SSP-3 sample studied by WGBS, and an average methylation gain of >10 fold over at least 5 CpGs (p-value <0.05) in all other SSP samples tested by methyl-array.

(c) The distance between the middle of region (EMR) and the TSS of neighboring gene.

(d) Fold change is calculated using methylation fraction values obtained from WGBS of SSP and GU samples (Fold change = SSP/GU).

### The SSP-specific DNA methylation pattern shows specific features

**Fig 4:**
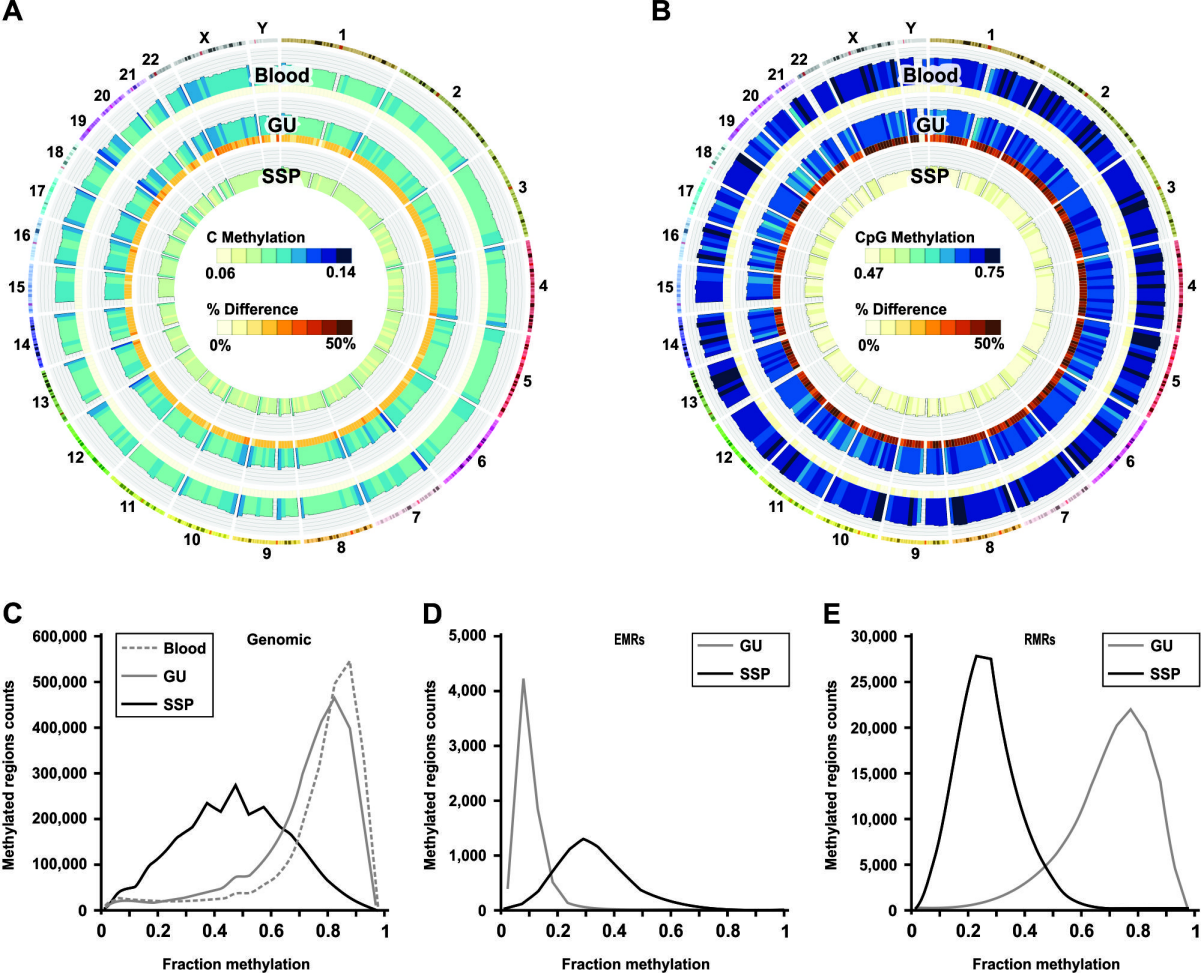
Global loss of methylation and partial methylation in SSP. **(A)** Global level of cytosine methylation in SSP, GU and blood. **(B)** Global level of CpG methylation in SSP, GU and blood. Histogram of fraction methylation in colon SSP, GU and blood **(C-E). (C)** In normal tissues most of the CpGs are either unmethylated or highly methylated (75–95% methylated). In SSP the very high level of methylation is lost and methylation level shows a more disperse distribution (35–80% methylated). **(D)** The EMRs show mostly 5–15% methylation in uninvolved colon tissue, while they are 15–45% methylated in SSP. **(E)** The RMRs in SSP were originally 65–90% methylated in uninvolved colon but this level is reduced to 15–40% in SSP.

For full characterization of the SSP-specific DNA methylation pattern, we applied the USeq package for comprehensive analysis of the SSP, GU and blood methylome datasets obtained from WGBS. The conversion rate was calculated about 99% for all three samples, and the sequencing covered the genome more than 36 fold. SSP genome shows a considerable level of global loss of methylation. The global level of cytosine methylation in blood and GU is 5.9% and 5.6% respectively. SSP genome has 45% reduction and shows 3.1% global cytosine methylation (Fig 4A). In the context of CpG dinucleotide the global level of methylation in blood, GU, and SSP is 74.7%, 70.0%, and 44.7% respectively which equals to 36.1% reduction in CpG methylation (Fig 4B). Comparing the SSP genome to GU genome retrieve about 7,000 regions that showed 3 to 30 fold increase in methylation (enriched methylated regions, EMRs), and 140,000 regions that showed 3 to 20 fold decrease in methylation (reduced methylated regions, RMRs) (S1 File).

The histogram of methylation fraction shows the genome in both blood and GU tissue contain either unmethylated or highly methylated regions (Fig 4C), while in SSP the genome harbor mostly regions with average level of methylation. The histogram from the methyl-array shows similar results (S2 Fig). The regions that gained methylation in SSP compared to GU show mostly 15–45% methylation in SSP while they were only 5–15% methylated in GU (Fig 4D), and the regions that lost methylation in SSP, were about 65–90% methylated in GU while they are only 15–40% methylated in SSP (Fig 4E). This is again showing that in SSP the level of methylation is getting close to the average level, as loss of methylation occurs at regions with high level of methylation, but gain of methylation happens at regions that had very low level of methylation normally, such as CGIs.

**Fig 5:**
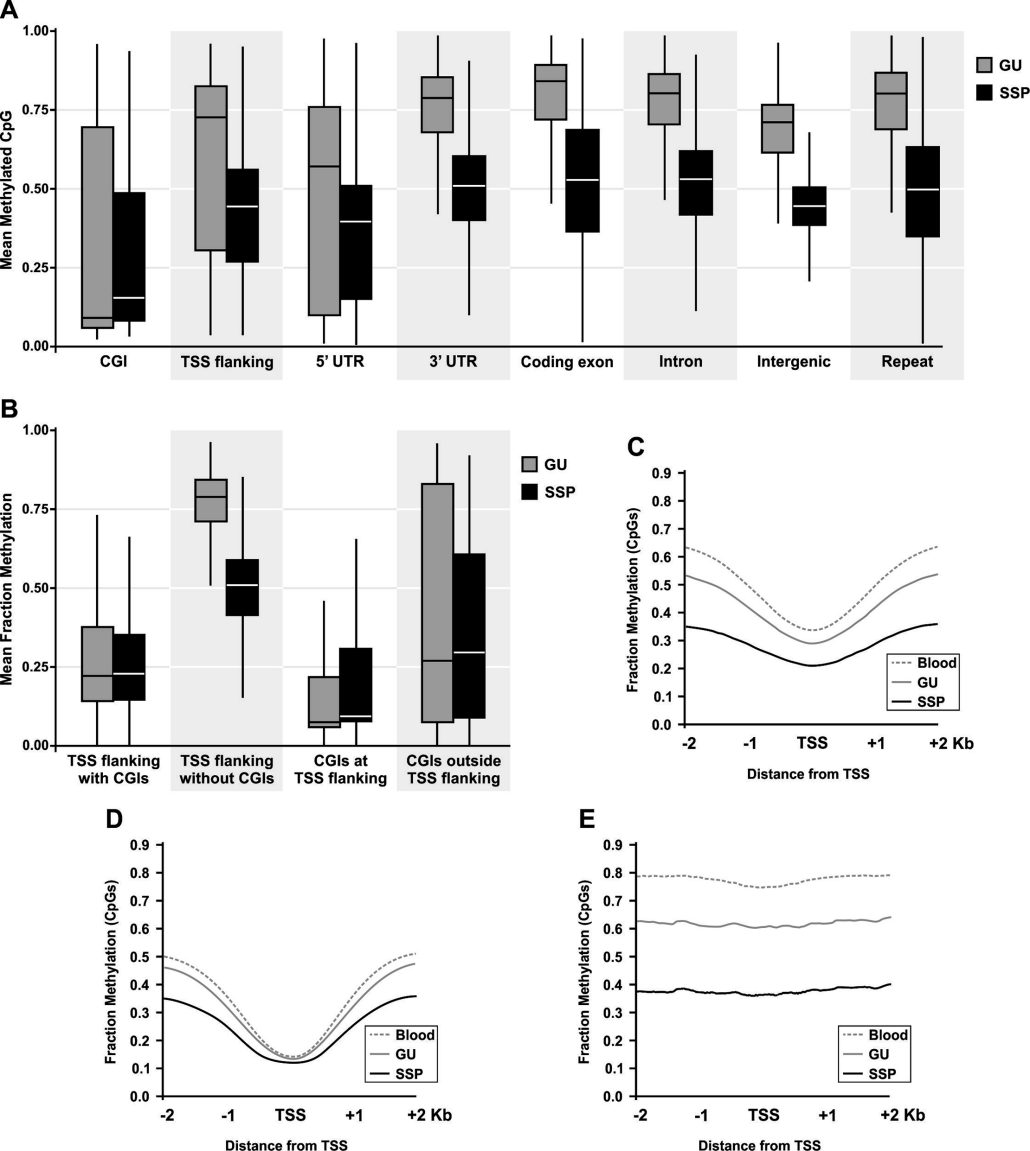
SSP-specific gain of methylation targets CGIs. **(A)** The global loss of methylation in SSP genome is detected all over the genome except at CGIs. The CGIs show the lowest level of DNA methylation in GU tissue, and gain methylation in the SSP tissue. The TSS 4Kb flanking regions and 5’UTRs show lower level of methylation loss compared to other regions of the genome. **(B)** TSS flanking regions that are located at CGIs have low level of CpG methylation. Although TSS flanking regions that are not part of CGIs show methylation level close to the genomic level. CGIs that are located at TSS flanking regions show lower level of methylation than CGIs that are not located around TSS. **(C)** Average of methylation on 4Kb TSS flanking regions of all Ensemble transcripts. **(D)** Average of methylation on 4Kb TSS flanking regions of Ensemble transcripts that include CGIs in that 4 Kb. **(E)** Average of methylation on 4Kb TSS flanking regions of Ensemble transcripts that do not include CGIs in that 4 Kb.

We assessed the level of CpG methylation in SSP and GU colon tissue at different genomic regions (Fig 5A). Loss of methylation is detected in all regions tested with variable level. The level of reduction in methylation of CpGs is 38.5% at intergenic regions, 36.8% at repeats, 35% at introns, 32.9% at coding exons, 28.9% at TSS 4 Kb flanking, and 9.4% at CGIs. CpG methylation at introns, 3’UTRs and intergenic regions are pretty close to the global level in GU and SSP. The methylation level at repeats is 79% in GU and 49.9% in SSP that is higher than the global level. The level of CpG methylation at coding exons is 58.5% in GU and 39.2% in SSP. In 5’UTR it is 65.7% in GU and 43.9% in SSP. In 4 Kb flanking regions around TSS of transcripts the CpG methylation is 50.7% in GU and 36% in SSP (We used Ensemble transcripts for this analysis. If the TSSs for Ensemble genes are considered this level will reach 26.6% and 21.8% for GU and SSP respectively). CpG islands show the lowest level of CpG methylation in the genome. They are 18% methylated in GU and 16.3% methylated in SSP.

Dissecting the TSS flanking regions to regions that have CGIs and the ones without CGIs shows that only when CGIs are present around TSS these regions have low methylation, as TSS flanking regions without CGIs are 72.3% methylated at CpGs in GU and 45% methylated in SSP (Fig 5B) which is close to the level detected in non-promoter regions. This level is only 29% at TSS flanking regions with CGI in GU and 23.7% in SSP. Although this shows that being part of CGIs dictate the low methylation level in normal state and lower level of methylation loss in SSP, dissecting CGIs to those that are at TSS flanking sites and those that are not around TSS shows that being around TSS reduces the level of methylation in CGIs (Fig 5B).

The RMRs are dispersed over the genome while they are retracted from CGIs (S3 and S4 Figs). Despite the RMRs, the EMRs are enriched at CGIs, and there is 12.6 or 14.79 fold enrichment of EMRs over CGIs whether or not they are located at TSS flanking regions. Therefore lower level of reduction in CpG methylation at CGIs and gene promoters drives from both lower level of methylation loss and higher level of methylation gain at those regions. EMRs are enriched 9.94 times at TSS flanking sites when there are CGIs present there, otherwise EMRs at TSS flanking happen in 0.46 of random frequency, while RMRs can localize there with 1.35 fold enrichment. The enrichment of EMRs at CGIs and the retraction of RMRs from CGIs are more pronounced as the condensation of CpGs increases at these regions. The pattern of CpG methylation in 4 Kb TSS flanking regions shows drop of methylation at TSS and increase of methylation as the distance from TSS increases. Both regions show loss of methylation in SSP, although the difference between methylation level of TSS and TSS flanking sites is reduced in SSP. However if we divide the Ensemble transcripts to those with CGI in the 4 Kb flanking region of TSS and those without CGI in 4 Kb TSS flanking region, these two groups of transcripts show very different methylation level around TSS (Fig 5D and 5E). The methylation at TSS of transcripts with CGI in 4 Kb flanking regions is about 13% in blood and GU, and 11% in SSP. This level increases as the distance from TSS increases (Fig 5D). While in transcripts that there is no CGI in 4 Kb TSS flanking regions, TSSs do not show significant low level of DNA methylation compared to the flanking regions and the rest of genome (Fig 5E).

### The correlation of BRAF-V600E mutation with gene expression profile is less pronounced, in comparison to its correlation with the DNA methylation pattern

**Fig 6:**
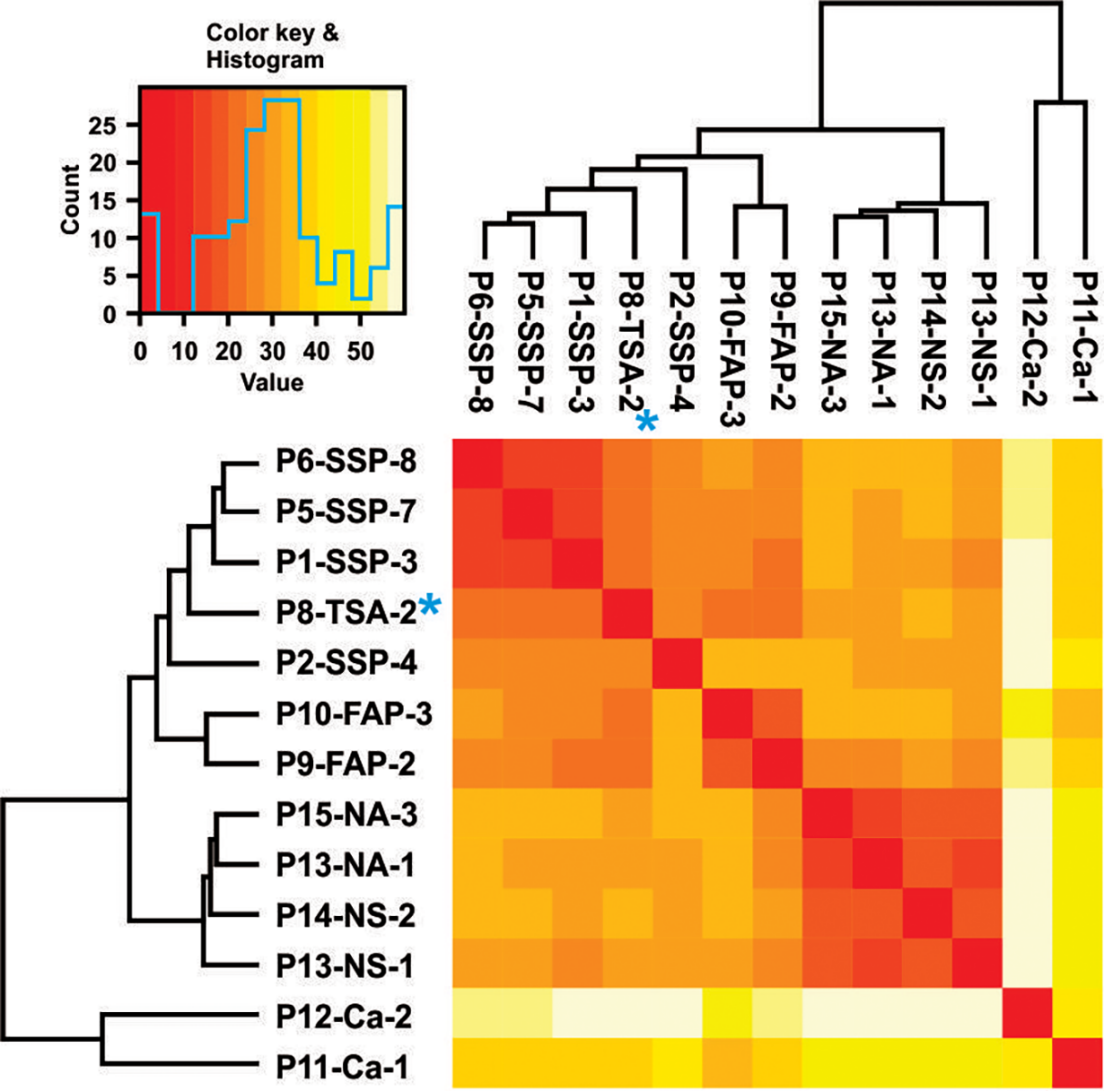
Gene expression profiling of colon samples. Hierarchical clustering and sample-to-sample distance heatmap of the expression of protein coding genes. SSP samples are clustered together, FAP samples also clustered together. Gene expression in TSA-2 that has both APC and BRAF mutation is more similar to the pattern of SSP expression cluster. The gene expression pattern of FAP show about 50% similarity to the gene expression of SSP, although the DNA methylation patterns of these two polyp types differ significantly. Therefore studying the DNA methylation marker can lead to detect SSP more specifically than studying the gene expression.

To study the effect of BRAF-V600E mutation on gene expression profile we performed high-throughput RNA sequencing (RNA-Seq) on BRAF-V600E positive and negative samples. The RNA-Seq was performed on all available samples, from the exact same list of samples we evaluated the DNA methylation for (see above). The sequencing datasets were analyzed using the USeq, DESeq and EdgeR packages, and hierarchical clustering was performed using distance matrix among samples. The SSP and FAP samples are each clustered together based on RNA expression (Fig 6), however, they show some similarities in the expression pattern of protein coding genes. In SSP 796 protein coding genes showed more than 2 fold up-regulation, and 1307 protein coding genes showed more than 2 fold down-regulation (FDR < 0.01). From these 400 and 486 protein-coding genes are up- or down-regulated, respectively, only in the SSPs and the rest show a similar trend in up- or down-regulation in both SSP and FAP samples. This remarkably differs from the DNA methylation differences between these two colon polyps (see above) (S2 File). Interestingly, while the hierarchical clustering of DNA methylation profiles grouped all BRAF-V600E positive samples together (Fig 1), clustering of the gene expression profiles did not result in such robust grouping (Fig 6). This indicates that the correlation of BRAF-V600E mutation with gene expression profile is less pronounced, in comparison to its correlation with the DNA methylation pattern.

The GO term analysis of the genes up-regulated only in SSP show enrichment for genes which encode extracellular proteins. Analysis of the function of these genes shows enrichment for the genes expressed in normal or tumor sate of stomach. TFF1 and TFF2 are both highly expressed in SSP. MUC6 is a gastric mucin that is highly over expressed in SSP. MUC17 is another mucin gene that is normally expressed in the intestine and is highly expressed in SSP. Other secretory proteins that are highly over expressed in SSP are SEMG1 and SEMG2. Both proteins are predominant in semen and have role in making gel matrix. Interestingly NACA2 is also up-regulated in SSP which may be partly incorporating to the mis-regulation of protein secretion in SSP. Among the genes down-regulated only in SSP there are many genes encoding ion channel or regulators of the channels, such as SCNN1G, CLIC6, CLCA1, KCNV1, KCNG3, KCNMA1, and SGK1. Another dominant group of genes that are down-regulated in SSP is encoding cell adhesion and junction proteins such as PCDH20, and GJD3. Interestingly DACT2 and WNK2 that are inhibitor of WNT signaling, and negative regulator of ERK/MAPK pathway respectively are both down-regulated in SSP.

## Discussion

While pathological examination of colon polyps provide valuable initial insights for screening and diagnosis, molecular features provide reliable markers for conclusive characterization of the polyps. Here a, combination of genome-wide mutation detection, DNA methylation analysis and transcriptome profiling on multiple normal and polyp samples revealed that: 1) the activation mutation BRAF-V600E is the sole recurring somatic mutation in SSPs; 2) there is a strong connection between BRAF-V600E mutation and a unique SSP-specific DNA methylation pattern; and 3) this unique DNA methylation profile introduces a panel of biomarkers with potential applicability for non-invasive early diagnosis and surveillance of SSP-related CRCs.

Although involvement of BRAF activating mutations in SSPs has been reported previously, those studies relied primarily on targeted mutation studies of specific DNA segments from limited number of oncogenes and tumor suppressor genes [2, 16, 17, 32]. This left open the possibly that other mutations might exist in addition to those know targets. Here, we demonstrated BRAF-V600E as the major mutation in SSPs using an unbiased comprehensive whole exome analysis of multiple SSP samples. It is important to note that exclusion of the involvement of uncommon background mutations (i.e. those not shared between different SSP samples) increases our confidence when connecting the other common molecular features (e.g. shared DNA methylation signatures) of SSPs to the single BRAF-V600E mutation. In addition, our whole genome DNA methylation profiling surveys revealed that while in normal colon samples about 70% of all CpGs are methylated, a significant global loss of methylation in SSP was evident with about 50% of all CpGs in all tested SSP samples. However, despite the global loss of DNA methylation, we detected significant gain of methylation reproducibly at specific CpG islands. Strikingly, all serrated polyp biopsies (with BRAF-V600E mutation) that were tested by WGBS showed a similar pattern of DNA methylation over the genome distinct from normal uninvolved colon or other samples such as FAPs (Fig 1A). In addition, the SSP-specific DNA methylation pattern showed a strong correlation with presence of BRAF-V600E mutation in the polyps. Hierarchical clustering of different samples, based on their DNA methylation patterns showed that all samples containing the BRAF-V600E mutation (i.e. all SSPs, one TSA and one carcinoma sample) clustered together. In a striking example, a single TSA sample was isolated from part of an adenomatous polyp from a FAP patient with mutation in the APC gene. The TSA portion of the polyp carried mutations in both BRAF and APC genes. Consistent with a strong link between the BRAF mutation and the coincident methylation changes only the BRAF-V600E positive portion of the polyp (the P8-TSA-2 sample), but not the APC mutant portion (the P8-FAP-1 sample) showed the SSP-specific DNA methylation pattern. Additionally while APC mutant adenomatous polyps showed small DNA methylation changes, the BRAF-V600E mutant polyps showed profound methylation changes compare to the normal DNA methylation status.

Previous studies have suggested expression based markers for classifying SSP [33]. Here, while our survey showed ~50% similarity between gene expression profiles of FAPs and SSPs, our comprehensive DNA methylation profiling on both sample series revealed a remarkable difference between these two types of colon polyps. Our results, therefore, show that DNA methylation is more reliable for conclusive characterization of SSPs. As a result a robust set of DNA methylation molecular markers, in the context of a panel of methylated fragments, are introduced here (Table 2), that could be used for characterization of SSPs in different analytical settings specially via non-invasive protocols such as fecal DNA screening[34–36].

Our working also directly links mutation in a major oncogene with specific consequences for remodeling the epigenome. It will be of great interest to understand how BRAF-V600E is connected to the formation of such unique SSP-specific DNA methylation pattern and what other factors are possibly required to fully develop and maintain this signature. It is possible that BRAF directly or indirectly regulates DNA methylation/demethylation effectors via downstream signaling pathways recruiting components of the chromatin modifying machinery. In addition, it is possible that this remodeled epigenome is permissive for further tumor development.

## Conclusions

The results presented here provide strong evidence that BRAF-V600E mutation is the main cause of generation of SSP and SSP-specific DNA methylation pattern. All three SSP separated from one patient (P1-SSP-1, P1-SSP-2, and P1-SSP-3) show BRAF-V600E mutation while this mutation was not detected in the GU portion of the colon of this patient, suggesting that the BRAF mutations happen independently in all three SSP. These SSP specific methylation patterns effectively distinguish SSP from adenomatous polyps which could be important for both diagnosis and treatment. It also suggests that BRAF-V600E mutation directly or indirectly results in the remodeling of the epigenome and that this may set a stage for tumor progression.

## Supporting information

S1 Fig Histopathological manifestation of SSP, TSA, and FAP samples. H&E stained sections of six samples are shown. Samples marked by asterisk (P8-FAP-1 and P8-TSA-2) are from two different portions of one polyp. The P8-FAP-1 was mutated in APC but not in BRAF, while P8-TSA-2 was confirmed to contain both APC and the BRAF-V600E mutations.
(PDF)

S2 Fig Histogram of methylated CpGs in SSP, TSA, FAP, carcinoma and normal samples. From the methyl array data the CpGs in FAP and normal samples are either unmethylated or highly methylated, making two peaks of fraction methylation, one close to zero and one above 0.9. However in SSPs, and BRAF mutant TSA and carcinoma samples both of these peaks are smaller and a third peak appeared around 0.3–0.6 methylation fraction. This confirms the partial methylation feature that is seen by WGBS of SSP compared to normal tissues.
(PDF)

S3 Fig CGIs show gain of methylation in SSP. There is about 10 fold enrichment of EMRs at TSS flanking regions (4Kb) that include CGIs while the fold enrichment of EMRS at TSS flanking regions without CGIs is less than 1. The RMRs occur at TSS flanking regions with CGIs with fold enrichment less than 1, while the occurrence of RMRs at TSS flanking regions without CGIs is detected with fold enrichment close to 1. EMRs are enriched at CGIs regardless of whether they are at TSS flanking or outside of TSS flanking regions (about 13 and 15 folds, respectively), while RMRs are excluded from these regions significantly.
(PDF)

S4 Fig Regional analysis of CpG methylation changes in SSP. **(a)** Gain of methylation in SSP is enriched at CGIs, while the loss of methylation is mostly at regions that are not CGI or CGI shore and shelf. **(b)** In SSP CpGs that show gain of methylation are enriched at promoter regions, while CpGs that show loss of methylation are retracted from promoter regions and are more localized at introns.
(PDF)

S1 Table Sequencing primers for BRAF and KRAS mutations. All samples are tested for BRAF-V600E and KRAS codon 12 and 13 mutations.
(PDF)

S2 Table Nested PCR primers for validation of EMRs. In the sequence columns “R” is either of A or G and “Y” is either of C or T nucleotides.
(PDF)

S3 Table Coverage of exome sequencing. **Eight Polyps from six patients were used for mutation study.** Blood DNA from four patients were available and used as control for somatic mutations in colon. Whole exome was captured by Agilent SureSelect Human All Exons 50Mb that target for about 51 Mb bases from whole exome. Captured exomes were sequenced by Illumina HiSeq 2000. Reads were aligned to hg19 by Novalign program. Total reads and reads on exome are listed. On average 92%, 80%, and 68% of the exome are covered with more than 10, 20, or 30 sequenced reads, respectively.
(PDF)

S4 Table Non-synonymous mutations found by exome sequencing. VARSCAN was used to find somatic and germline mutations in each polyp-blood pair compared to hg19 with minimum variation frequency of 5%. Blood data of patient #4 was used as the paired sample for SSP-7 and SSP-8 in VARSCAN analysis. Each sample showed about 17K-30K non-synonymous mutations in exonic and splicing regions, which equals to about 277–485 mutations per megabase.
(PDF)

S5 Table BRAF-V600E is the only recurrent somatic mutation in SSP. From the eight exome sequenced SSP samples the only common somatic mutation in any combination of 4 or more of the samples was BRAF-V600E mutation.
(PDF)

S1 File EMRS with significant gain of methylation in SSP. This table reports the enhanced methylated regions (EMRs) with more than 10 fold gain of methylation in SSP compared to GU (p-value <0.001).
(XLSX)

S2 File Gene expression changes in SSP. This table reports the genes with expression changes in SSP (fold change >2, p-value <0.01).
(XLSX)

S3 File Supplementatry information. This file contains all the supplementary figures and tables and their legends in a single PDF file.
(PDF)
