# Supplementary figures and images for: Active BRAF-V600E is the key player in generation of a sessile serrated polyp-specific DNA methylation profile
Source: PLoS One. 2018 Mar 28;13(3):e0192499. doi: 10.1371/journal.pone.0192499 (PMC5873940; doi:10.1371/journal.pone.0192499)

**P1-SSP-3**

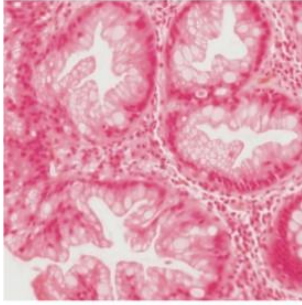

**P7-TSA-1**

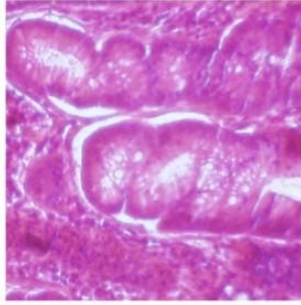

**P8-FAP-1\***

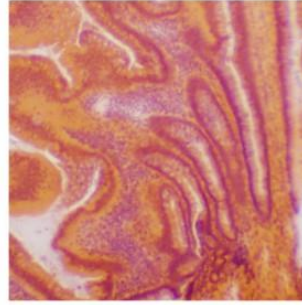

**P7-SSP-9**

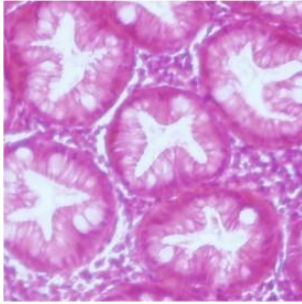

**P8-TSA-2\***

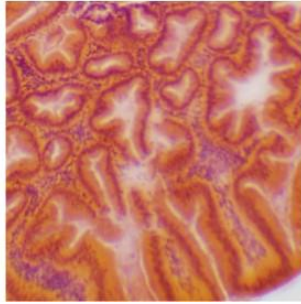

**P9-FAP-2**

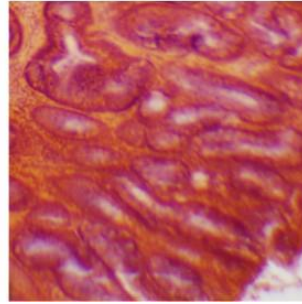

Supplement: S1 Fig — H&E stained sections of six samples are shown. Samples marked by asterisk (P8-FAP-1 and P8-TSA-2) are from two different portions of one polyp. The P8-FAP-1 was mutated in APC but not in BRAF, while P8-TSA-2 was confirmed to contain both APC and the BRAF-V600E mutations. (PDF) [file pone.0192499.s001.pdf]

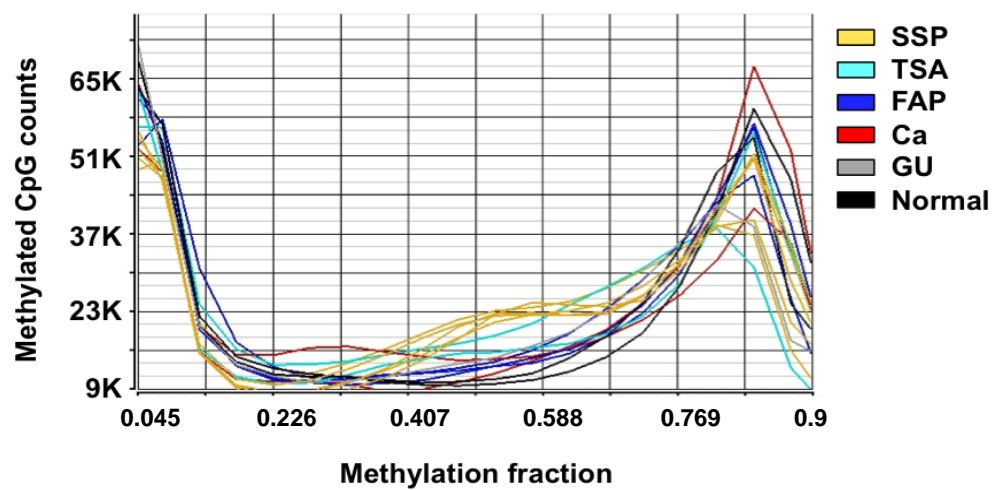

Supplement: S2 Fig — From the methyl array data the CpGs in FAP and normal samples are either unmethylated or highly methylated, making two peaks of fraction methylation, one close to zero and one above 0.9. However in SSPs, and BRAF mutant TSA and carcinoma samples both of these peaks are smaller and a third peak appeared around 0.3–0.6 methylation fraction. This confirms the partial methylation feature that is seen by WGBS of SSP compared to normal tissues. (PDF) [file pone.0192499.s002.pdf]

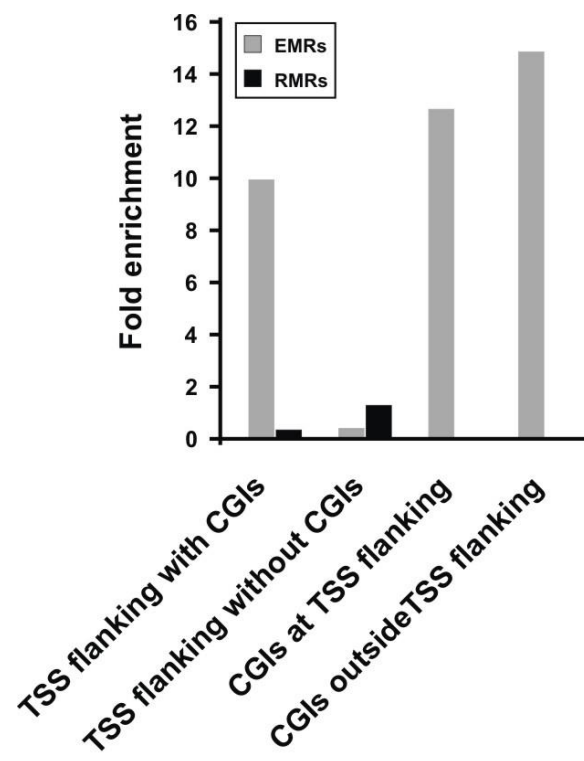

Supplement: S3 Fig — There is about 10 fold enrichment of EMRs at TSS flanking regions (4Kb) that include CGIs while the fold enrichment of EMRS at TSS flanking regions without CGIs is less than 1. The RMRs occur at TSS flanking regions with CGIs with fold enrichment less than 1, while the occurrence of RMRs at TSS flanking regions without CGIs is detected with fold enrichment close to 1. EMRs are enriched at CGIs regardless of whether they are at TSS flanking or outside of TSS flanking regions (about 13 and 15 folds, respectively), while RMRs are excluded from these regions significantly. (PDF) [file pone.0192499.s003.pdf]

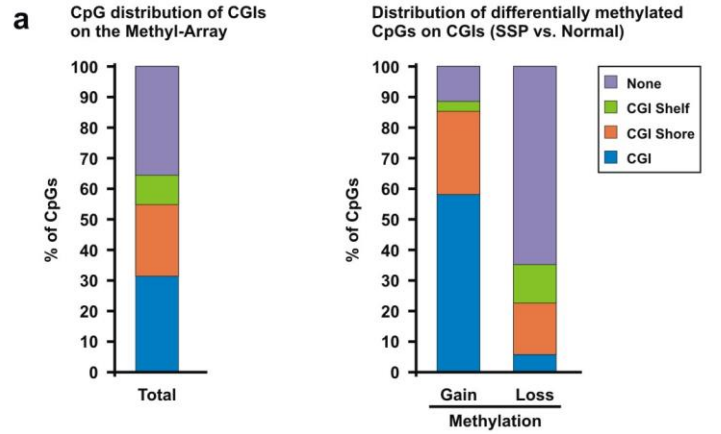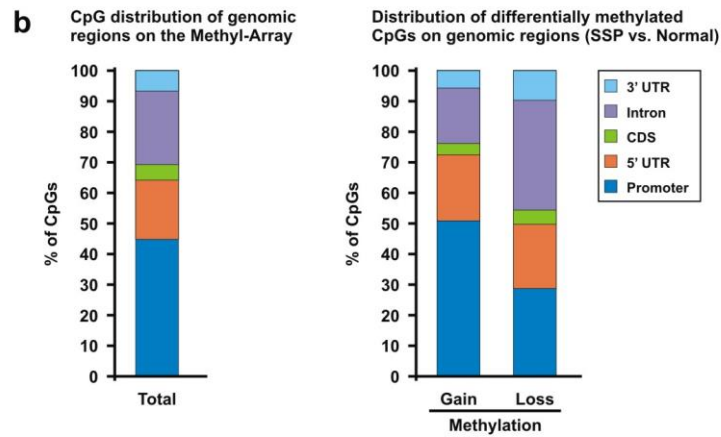

Supplement: S4 Fig — (a) Gain of methylation in SSP is enriched at CGIs, while the loss of methylation is mostly at regions that are not CGI or CGI shore and shelf. (b) In SSP CpGs that show gain of methylation are enriched at promoter regions, while CpGs that show loss of methylation are retracted from promoter regions and are more localized at introns. (PDF) [file pone.0192499.s004.pdf]
