# Supplementary material for: Active BRAF-V600E is the key player in generation of a sessile serrated polyp-specific DNA methylation profile
Source: PLoS One. 2018 Mar 28;13(3):e0192499. doi: 10.1371/journal.pone.0192499 (PMC5873940; doi:10.1371/journal.pone.0192499)
Supplement: S1 Table — All samples are tested for BRAF-V600E and KRAS codon 12 and 13 mutations. (PDF) [file pone.0192499.s005.pdf]

Gene	Primer	Sequence (5’ to 3’)
BRAF	BRAF-F	CTATAAACTTAGGAAAGCATCTCACCT
	BRAF-R	GATTTTTGTGAATACTGGGAACTATG
KRAS	KRAS-F	TATAAGGCCTGCTGAAAATGACT
	KRAS-R	TACTCATGAAAATGGTCAGAGAAAC
