# Supplementary material for: Active BRAF-V600E is the key player in generation of a sessile serrated polyp-specific DNA methylation profile
Source: PLoS One. 2018 Mar 28;13(3):e0192499. doi: 10.1371/journal.pone.0192499 (PMC5873940; doi:10.1371/journal.pone.0192499)
Supplement: S2 Table — In the sequence columns “R” is either of A or G and “Y” is either of C or T nucleotides. (PDF) [file pone.0192499.s006.pdf]

Gene	1st PCR-Primer	Sequence (5' to 3')	2nd PCR-Primer	Sequence (5' to 3')
**MAP6**	MAP6-F1	GATGGTTTTTTAGTTTTGTTAGGTGG	MAP6-F2	TATTAGGAYGTYGGTAGGAAGGAG
	MAP6-R1	CCRACCTCCTCTTTCTTCTTATAATTC	MAP6-R2	AAATCTCTATAATCTTCCTTCAACCTCC
**EOMES**	EOMES-F1	TTAGYGTGTGAGTTTGGGAGGAG	EOMES-F2	GGGGGTTTTGAGGAGTAAGAGG
	EOMES-R1	CTCACCAAAAACTACTCCCCTAACTACATAC	EOMES-R2	ACRACAACTAACTACTTCCTCTCCTCC
**ARHGAP20**	ARHGAP20-F1	AAGAGGTTAGGYGATGTTGTGG	ARHGAP20-F2	TGTTGTGGGTTTTTAAATTTGTATAGTTG
	ARHGAP20-R1	TCAACCCTCCTCCAACCTAAAC	ARHGAP20-R2	TCCAACCTAAACCCTCCTAACCTC
**CNTFR**	CNTFR-F1	GGTGGGGTYGATTGTGGATTAG	CNTFR-F2	GATTGTGGATTAGAGGGAGGTG
	CNTFR-R1	CAAACTCRCACAAACCAAAAAAACTTAC	CNTFR-R2	ACCAAAAAAACTTACAAAAAAACACACT
**PITX2**	PITX2-F1	AAAAGTGAATGTGTYGTTGTAGTGAG	PITX2-F2	ATTTATATTTGYGTTTGTATATTTTATAGG
	PITX2-R1	CCAAACTCCATACTAACTCCTACCC	PITX2-R2	AATTAATCCACACAACAATTTCTTC
**CALCA**	CALCA-F1	TAAAGAGYAGGYAGGTGTGAYAGTG	CALCA-F2	GGYYAGAAGAGTATYYTGAGGTG
	CALCA-R1	CCTARRRRCTAATTTCTACTCTACCTC	CALCA-R2	AARCTTCTTTCTTRCCACTCTRRAC
**CHFR**	CHFR-F1	GGGGATGAYTYYTAGGAGTAGAAG	CHFR-F2	AGATYYGAAGTYTGAGGYAYAGGG
	CHFR-R1	ARRTCTCARRCTAATCTCRAACTCCT	CHFR-R2	TCCTACCTARRCCTTCCAAARCAC
**TRANK1**	TRANK1-F1	GGAGGGTYYGYAYYAGGAYYG	TRANK1-F2	GGGAAGYGYTTYYYTGTGGGYAGG
	TRANK1-R1	RRCCGATCACTTTCTTTACCTCC	TRANK1-R2	ATTCRAARCACTCRTTTATTCAAA
**MLH1**	MLH1-F1	AAGGYAAGAGAATAGGYTTTAAAGT	MLH1-F2	TGYYTTGTGATATYTGGAGATAAGT
	MLH1-R1	CTTRCRRCCTTTCTAACRTT	MLH1-R2	RACRCCCAAAARAARCAARAT
**WNT3A**	WNT3A-F1	TAATYYGATAATAATTTTTYTYTTYG	WNT3A-F2	TYTYTTYGAGATGGTTYAGGAG
	WNT3A-R1	RTCCATTCARRRTARAACACA	WNT3A-R2	RCCRACAARACAAARATCCTA
**WNT5A**	WNT5A-F1	ATYGGYTYGTAAAYTGATTATG	WNT5A-F2	TGAAAYATAYGATGTTAATTYGGA
	WNT5A-R1	CCTCTCARATAATTTTCAARCATAC	WNT5A-R2	AATTTTCAARCATACAARTTTAAACAAC
**ATP2B4**	ATP2B4-F1	GGAGGYTYAGAGTGYAGYTATT	ATP2B4-F2	YYTATTTTYAGTAATYTGATTAGGGGT
	ATP2B4-R1	CTRTRCTCCTTTRACTTRACCT	ATP2B4-R2	CTTTRACTTRACCTTRAATCTARARAC
