# Supplementary material for: Active BRAF-V600E is the key player in generation of a sessile serrated polyp-specific DNA methylation profile
Source: PLoS One. 2018 Mar 28;13(3):e0192499. doi: 10.1371/journal.pone.0192499 (PMC5873940; doi:10.1371/journal.pone.0192499)
Supplement: S3 Table — Eight Polyps from six patients were used for mutation study. Blood DNA from four patients were available and used as control for somatic mutations in colon. Whole exome was captured by Agilent SureSelect Human All Exons 50Mb that target for about 51 Mb bases from whole exome. Captured exomes were sequenced by Illumina HiSeq 2000. Reads were aligned to hg19 by Novalign program. Total reads and reads on exome are listed. On average 92%, 80%, and 68% of the exome are covered with more than 10, 20, or 30 sequenced reads, respectively. (PDF) [file pone.0192499.s007.pdf]

Samples	Duplicate Removed Sequenced Bases	Sequenced Bases On Targeted Exome	Percent Of Exome Covered By More Than 10 Reads	Percent Of Exome Covered By More Than 20 Reads	Percent Of Exome Covered By More Than 30 Reads
P1-SSP-1	10,854,821,908	6,606,504,067	94.86%	91.76%	88.56%
P1-SSP-2	8,299,845,447	5,022,503,154	93.76%	89.54%	84.81%
P1-SSP-3	10,979,531,148	6,698,198,231	94.14%	90.66%	87.30%
P1-Blood	9,808,054,926	5,873,064,354	93.07%	88.57%	84.15%
P2-SSP-4	4,769,070,716	2,157,812,673	91.92%	74.07%	55.69%
P2-Blood	6,138,952,965	2,826,277,707	95.24%	83.31%	68.77%
P3-SSP-5	6,583,625,990	2,778,674,249	86.76%	70.60%	57.96%
P3-Blood	5,355,378,742	2,397,771,499	95.14%	82.10%	65.76%
P4-SSP-6	4,763,784,363	2,106,901,779	89.60%	70.90%	53.38%
P4-Blood	6,178,515,234	2,619,831,203	95.84%	84.35%	69.45%
P5-SSP-7	4,352,425,949	1,978,459,326	90.12%	70.19%	51.03%
P6-SSP-8	4,388,662,394	2,017,750,565	90.62%	70.95%	51.82%
