# Supplementary material for: Active BRAF-V600E is the key player in generation of a sessile serrated polyp-specific DNA methylation profile
Source: PLoS One. 2018 Mar 28;13(3):e0192499. doi: 10.1371/journal.pone.0192499 (PMC5873940; doi:10.1371/journal.pone.0192499)
Supplement: S4 Table — VARSCAN was used to find somatic and germline mutations in each polyp-blood pair compared to hg19 with minimum variation frequency of 5%. Blood data of patient #4 was used as the paired sample for SSP-7 and SSP-8 in VARSCAN analysis. Each sample showed about 17K-30K non-synonymous mutations in exonic and splicing regions, which equals to about 277–485 mutations per megabase. (PDF) [file pone.0192499.s008.pdf]

Paired Analysis	Total Mutation	SNP	Indel	Somatic	Germline	Homozygote	Heterozygote
P1-SSP-1 vs P1-Blood	16,941	16,565	376	3,602	13,339	4,220	12,721
P1-SSP-2 vs P1-Blood	19,020	18,638	382	5,679	13,341	4,233	14,787
P1-SSP-3 vs P1-Blood	16,403	16,043	360	3,148	13,255	4,192	12,211
P2-SSP-4 vs P2-Blood	29,666	29,162	504	17,164	12,502	4,416	25,250
P3-SSP-5 vs P3-Blood	22,556	22,128	428	10,967	11,589	4,302	18,254
P4-SSP-6 vs P4-Blood	19,460	19,037	423	7,601	11,859	4,398	15,062
P5-SSP-7 vs P4-Blood	22,086	21,613	473	13,702	8,384	4,541	17,545
P6-SSP-8 vs P4-Blood	20,031	19,574	457	11,440	8,591	4,646	15,385
