# Supplementary material for: Active BRAF-V600E is the key player in generation of a sessile serrated polyp-specific DNA methylation profile
Source: PLoS One. 2018 Mar 28;13(3):e0192499. doi: 10.1371/journal.pone.0192499 (PMC5873940; doi:10.1371/journal.pone.0192499)
Supplement: S5 Table — From the eight exome sequenced SSP samples the only common somatic mutation in any combination of 4 or more of the samples was BRAF-V600E mutation. (PDF) [file pone.0192499.s009.pdf]

Samples	PCR-Confirmed Filter Passed Non-Synonymous Somatic Mutations
All 8 SSPs	BRAF-V600E
Any combination of 7 SSP samples	BRAF-V600E
Any combination of 6 SSP samples	BRAF-V600E
Any combination of 5 SSP samples	BRAF-V600E
Any combination of 4 SSP samples	BRAF-V600E
