# Supplementary material for: Active BRAF-V600E is the key player in generation of a sessile serrated polyp-specific DNA methylation profile
Source: PLoS One. 2018 Mar 28;13(3):e0192499. doi: 10.1371/journal.pone.0192499 (PMC5873940; doi:10.1371/journal.pone.0192499)
Supplement: S3 File — This file contains all the supplementary figures and tables and their legends in a single PDF file. (PDF) [file pone.0192499.s012.pdf]

SUPPLEMENTARY INFORMATION

Active BRAF-V600E is the key player in generation of a sessile serrated polyp-specific DNA methylation profile

Somaye Dehghanizadeh[1], Vahid Khoddami[2], Timothy L. Mosbruger[3], Sue S. Hammoud[4], Kornelia Edes[3], Therese S. Berry[3], Michelle Done[3], Wade S. Samowitz[5], James A. DiSario[6], Daniel G. Luba[6], Randall W. Burt[3,7] and David A. Jones[8]*

1 Department of Oncological Sciences, Huntsman Cancer Institute, University of Utah School of Medicine, Salt Lake City, UT, USA
2 Department of Cell Biology, Harvard Medical School, Boston, MA, USA
3 Huntsman Cancer Institute, University of Utah School of Medicine, Salt Lake City, UT, USA
4 Department of Human Genetics, University of Michigan Medical School, Ann Arbor, MI, USA
5 Department of Pathology, University of Utah School of Medicine, Salt Lake City, UT, USA
6 The Monterey Bay Gastroenterology Research Institute, Monterey, CA, USA
7 Department of Internal Medicine, University of Utah School of Medicine, Salt Lake City, UT, USA
8 Functional and Chemical Genomics, Oklahoma Medical Research Foundation, Oklahoma City, OK, USA
*Correspondence should be addressed to D. J. (David-Jones@omrf.org)

SUPPLEMENTARY FIGURES:
S1 Fig. Histopathological manifestation of SSP, TSA, and FAP samples.
S2 Fig. Histogram of methylated CpGs in SSP, TSA, FAP, carcinoma and normal samples.
S3 Fig. CGIs show gain of methylation in SSP.
S4 Fig. Regional analysis of CpG methylation changes in SSP.

SUPPLEMENTARY TABLES:
S1 Table. Sequencing primers for BRAF and KRAS mutations.
S2 Table. Nested PCR primers for validation of EMRs.
S3 Table. BRAF-V600E is the only recurrent somatic mutation in SSP.
S4 Table. Coverage of exome sequencing.
S5 Table. Non-synonymous mutations found by exome sequencing.

SUPPLEMENTARY FIGURES:

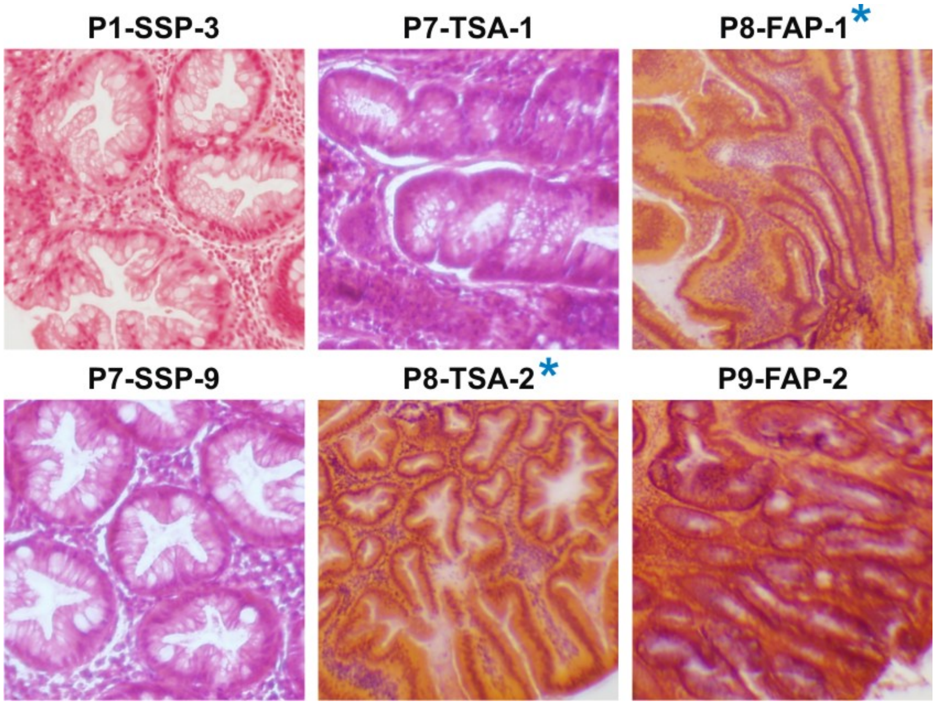

S1 Fig. Histopathological manifestation of SSP, TSA, and FAP samples. H&E stained sections of six samples are shown. Samples marked by asterisk (P8-FAP-1 and P8-TSA-2) are from two different portions of one polyp. The P8-FAP-1 was mutated in APC but not in BRAF, while P8-TSA-2 was confirmed to contain both APC and the BRAF-V600E mutations.

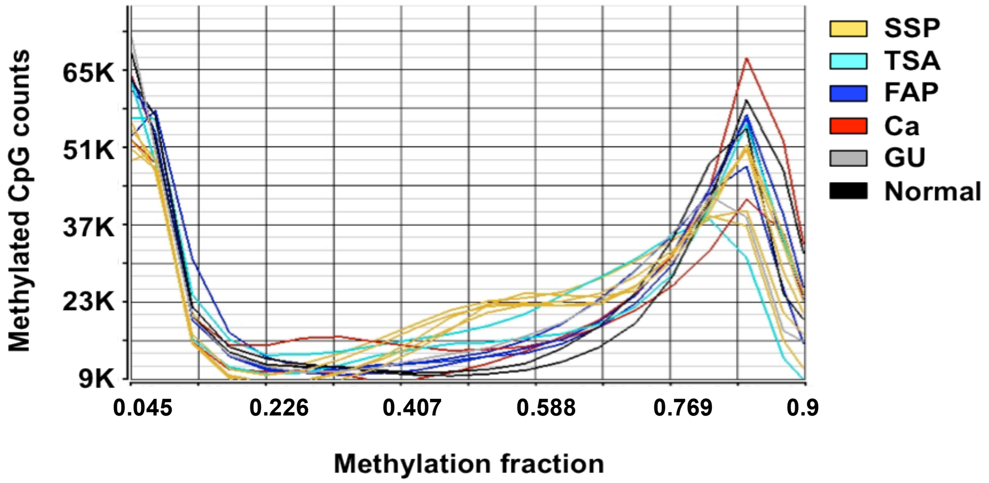

S2 Fig. Histogram of methylated CpGs in SSP, TSA, FAP, carcinoma and normal samples. From the methyl array data the CpGs in FAP and normal samples are either unmethylated or highly methylated, making two peaks of fraction methylation, one close to zero and one above 0.9. However in SSPs, and BRAF mutant TSA and carcinoma samples both of these peaks are smaller and a third peak appeared around 0.3-0.6 methylation fraction. This confirms the partial methylation feature that is seen by WGBS of SSP compared to normal tissues.

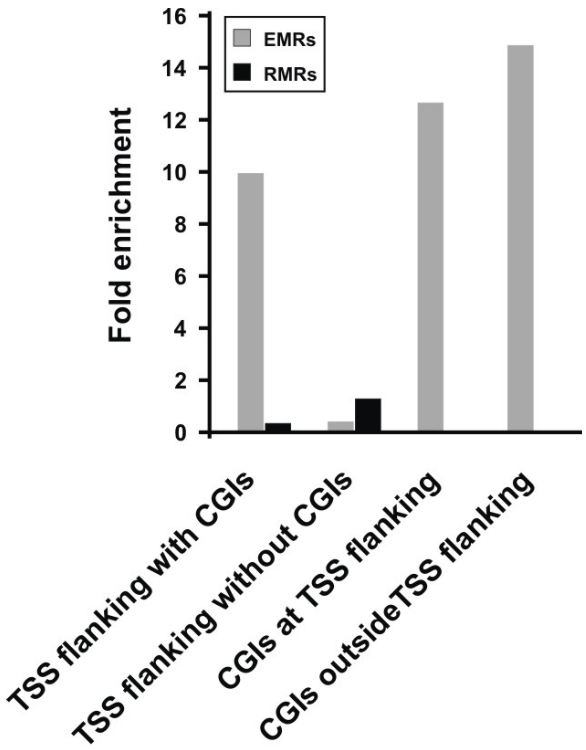

S3 Fig. CGIs show gain of methylation in SSP. There is about 10 fold enrichment of EMRs at TSS flanking regions (4Kb) that include CGIs while the fold enrichment of EMRS at TSS flanking regions without CGIs is less than 1. The RMRs occur at TSS flanking regions with CGIs with fold enrichment less than 1, while the occurrence of RMRs at TSS flanking regions without CGIs is detected with fold enrichment close to 1. EMRs are enriched at CGIs regardless of whether they are at TSS flanking or outside of TSS flanking regions (about 13 and 15 folds, respectively), while RMRs are excluded from these regions significantly.

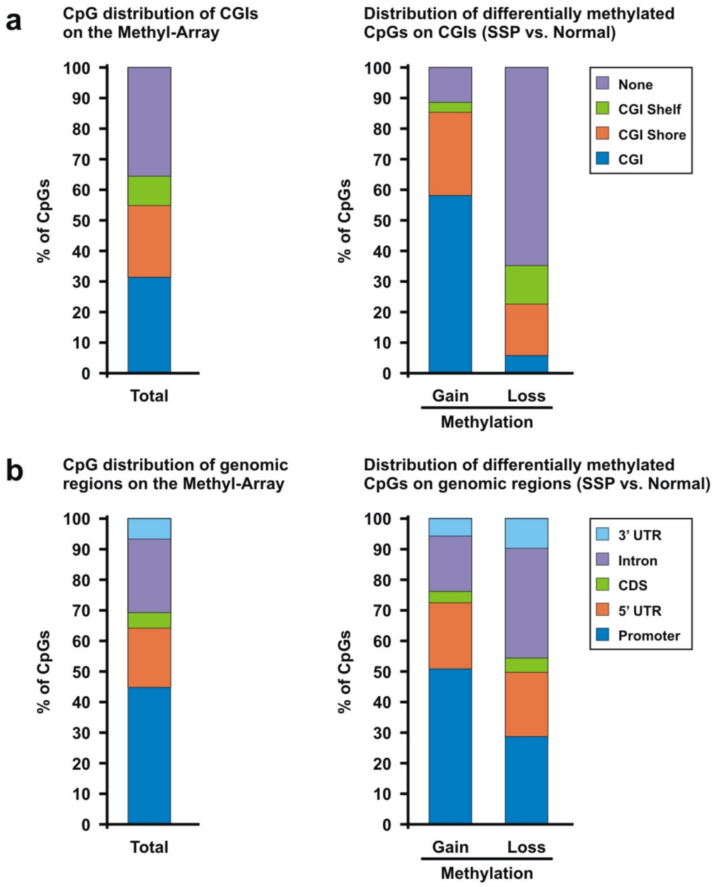

S4 Fig. Regional analysis of CpG methylation changes in SSP. (a) Gain of methylation in SSP is enriched at CGIs, while the loss of methylation is mostly at regions that are not CGI or CGI shore and shelf. **(b)** In SSP CpGs that show gain of methylation are enriched at promoter regions, while CpGs that show loss of methylation are retracted from promoter regions and are more localized at introns.

SUPPLEMENTARY TABLES:

Gene	Primer	Sequence (5' to 3')
**BRAF**	BRAF-F	CTATAAACTTAGGAAAGCATCTCACCT
	BRAF-R	GATTTTTGTGAATACTGGGAACTATG
**KRAS**	KRAS-F	TATAAGGCCTGCTGAAAATGACT
	KRAS-R	TACTCATGAAAATGGTCAGAGAAAC

S1 Table. Sequencing primers for BRAF and KRAS mutations. All samples are tested for BRAF-V600E and KRAS codon 12 and 13 mutations.

Gene	1st PCR-Primer	Sequence (5' to 3')	2nd PCR-Primer	Sequence (5' to 3')
**MAP6**	MAP6-F1	GATGGTTTTTTAGTTTTGTTAGGTGG	MAP6-F2	TATTAGGAYGTYGGTAGGAAGGAG
	MAP6-R1	CCRACCTCCTCTTTCTTCTTATAATTC	MAP6-R2	AAATCTCTATAATCTTCCTTCAACCTCC
**EOMES**	EOMES-F1	TTAGYGTGTGAGTTTGGGAGGAG	EOMES-F2	GGGGGTTTTGAGGAGTAAGAGG
	EOMES-R1	CTCACCAAAAACTACTCCCCTAACTACATAC	EOMES-R2	ACRACAACTAACTACTTCCTCTCCTCC
**ARHGAP20**	ARHGAP20-F1	AAGAGGTTAGGYGATGTTGTGG	ARHGAP20-F2	TGTTGTGGGTTTTTAAATTTGTATAGTTG
	ARHGAP20-R1	TCAACCCTCCTCCAACCTAAAC	ARHGAP20-R2	TCCAACCTAAACCCTCCTAACCTC
**CNTFR**	CNTFR-F1	GGTGGGGTYGATTGTGGATTAG	CNTFR-F2	GATTGTGGATTAGAGGGAGGTG
	CNTFR-R1	CAAACTCRCACAAACCAAAAAAACTTAC	CNTFR-R2	ACCAAAAAAACTTACAAAAAAACACACT
**PITX2**	PITX2-F1	AAAAGTGAATGTGTYGTTGTAGTGAG	PITX2-F2	ATTTATATTTGYGTTTGTATATTTTATAGG
	PITX2-R1	CCAAACTCCATACTAACTCCTACCC	PITX2-R2	AATTAATCCACACAACAATTTCTTC
**CALCA**	CALCA-F1	TAAAGAGYAGGYAGGTGTGAYAGTG	CALCA-F2	GGYYAGAAGAGTATYYTGAGGTG
	CALCA-R1	CCTARRRRCTAATTTCTACTCTACCTC	CALCA-R2	AARCTTCTTTCTTRCCACTCTRRAC
**CHFR**	CHFR-F1	GGGGATGAYTYYTAGGAGTAGAAG	CHFR-F2	AGATYYGAAGTYTGAGGYAYAGGG
	CHFR-R1	ARRTCTCARRCTAATCTCRAACTCCT	CHFR-R2	TCCTACCTARRCCTTCCAAARCAC
**TRANK1**	TRANK1-F1	GGAGGGTYYGYAYYAGGAYYG	TRANK1-F2	GGGAAGYGYTTYYYTGTGGGYAGG
	TRANK1-R1	RRCCGATCACTTTCTTTACCTCC	TRANK1-R2	ATTCRAARCACTCRTTTATTCAAA
**MLH1**	MLH1-F1	AAGGYAAGAGAATAGGYTTTAAAGT	MLH1-F2	TGYYTTGTGATATYTGGAGATAAGT
	MLH1-R1	CTTRCRRCCTTTCTAACRTT	MLH1-R2	RACRCCCAAAARAARCAARAT
**WNT3A**	WNT3A-F1	TAATYYGATAATAATTTTTYTYTTYG	WNT3A-F2	TYTYTTYGAGATGGTTYAGGAG
	WNT3A-R1	RTCCATTCARRRTARAACACA	WNT3A-R2	RCCRACAARACAAARATCCTA
**WNT5A**	WNT5A-F1	ATYGGYTYGTAAAYTGATTATG	WNT5A-F2	TGAAAYATAYGATGTTAATTYGGA
	WNT5A-R1	CCTCTCARATAATTTTCAARCATAC	WNT5A-R2	AATTTTCAARCATACAARTTTAAACAAC
**ATP2B4**	ATP2B4-F1	GGAGGYTYAGAGTGYAGYTATT	ATP2B4-F2	YYTATTTTYAGTAATYTGATTAGGGGT
	ATP2B4-R1	CTRTRCTCCTTTRACTTRACCT	ATP2B4-R2	CTTTRACTTRACCTTRAATCTARARAC

S2 Table. Nested PCR primers for validation of EMRs. In the sequence columns "R" is either of A or G and "Y" is either of C or T nucleotides.

Samples	Duplicate Removed Sequenced Bases	Sequenced Bases On Targeted Exome	Percent Of Exome Covered By More Than 10 Reads	Percent Of Exome Covered By More Than 20 Reads	Percent Of Exome Covered By More Than 30 Reads
**P1-SSP-1**	10,854,821,908	6,606,504,067	94.86%	91.76%	88.56%
**P1-SSP-2**	8,299,845,447	5,022,503,154	93.76%	89.54%	84.81%
**P1-SSP-3**	10,979,531,148	6,698,198,231	94.14%	90.66%	87.30%
**P1-Blood**	9,808,054,926	5,873,064,354	93.07%	88.57%	84.15%
**P2-SSP-4**	4,769,070,716	2,157,812,673	91.92%	74.07%	55.69%
**P2-Blood**	6,138,952,965	2,826,277,707	95.24%	83.31%	68.77%
**P3-SSP-5**	6,583,625,990	2,778,674,249	86.76%	70.60%	57.96%
**P3-Blood**	5,355,378,742	2,397,771,499	95.14%	82.10%	65.76%
**P4-SSP-6**	4,763,784,363	2,106,901,779	89.60%	70.90%	53.38%
**P4-Blood**	6,178,515,234	2,619,831,203	95.84%	84.35%	69.45%
**P5-SSP-7**	4,352,425,949	1,978,459,326	90.12%	70.19%	51.03%
**P6-SSP-8**	4,388,662,394	2,017,750,565	90.62%	70.95%	51.82%

S3 Table. Coverage of exome sequencing. Eight Polyps from six patients were used for mutation study. Blood DNA from four patients were available and used as control for somatic mutations in colon. Whole exome was captured by Agilent SureSelect Human All Exons 50Mb that target for about 51 Mb bases from whole exome. Captured exomes were sequenced by Illumina HiSeq 2000. Reads were aligned to hg19 by Novalign program. Total reads and reads on exome are listed. On average 92%, 80%, and 68% of the exome are covered with more than 10, 20, or 30 sequenced reads, respectively.

Paired Analysis	Total Mutation	SNP	Indel	Somatic	Germline	Homozygote	Heterozygote
**P1-SSP-1 vs P1-Blood**	16,941	16,565	376	3,602	13,339	4,220	12,721
**P1-SSP-2 vs P1-Blood**	19,020	18,638	382	5,679	13,341	4,233	14,787
**P1-SSP-3 vs P1-Blood**	16,403	16,043	360	3,148	13,255	4,192	12,211
**P2-SSP-4 vs P2-Blood**	29,666	29,162	504	17,164	12,502	4,416	25,250
**P3-SSP-5 vs P3-Blood**	22,556	22,128	428	10,967	11,589	4,302	18,254
**P4-SSP-6 vs P4-Blood**	19,460	19,037	423	7,601	11,859	4,398	15,062
**P5-SSP-7 vs P4-Blood**	22,086	21,613	473	13,702	8,384	4,541	17,545
**P6-SSP-8 vs P4-Blood**	20,031	19,574	457	11,440	8,591	4,646	15,385

S4 Table. Non-synonymous mutations found by exome sequencing. VARSCAN was used to find somatic and germline mutations in each polyp-blood pair compared to hg19 with minimum variation frequency of 5%. Blood data of patient #4 was used as the paired sample for SSP-7 and SSP-8 in VARSCAN analysis. Each sample showed about 17K-30K non-synonymous mutations in exonic and splicing regions, which equals to about 277-485 mutations per megabase.

Samples	PCR-confirmed filter-passed non-synonymous somatic mutations
All 8 SSPs	BRAF-V600E
Any combination o 7 SSPs	BRAF-V600E
Any combination o 6 SSPs	BRAF-V600E
Any combination o 5 SSPs	BRAF-V600E
Any combination o 4 SSPs	BRAF-V600E

S5 Table. BRAF-V600E is the only recurrent somatic mutation in SSP. From the eight exome sequenced SSP samples the only common somatic mutation in any combination of 4 or more of the samples was BRAF-V600E mutation.
